# Oligodendrocyte myelin glycoprotein as a novel target for pathogenic autoimmunity in the CNS

**DOI:** 10.1186/s40478-020-01086-2

**Published:** 2020-11-30

**Authors:** Ramona Gerhards, Lena Kristina Pfeffer, Jessica Lorenz, Laura Starost, Luise Nowack, Franziska S. Thaler, Miriam Schlüter, Heike Rübsamen, Caterina Macrini, Stephan Winklmeier, Simone Mader, Mattias Bronge, Hans Grönlund, Regina Feederle, Hung-En Hsia, Stefan F. Lichtenthaler, Juliane Merl-Pham, Stefanie M. Hauck, Tanja Kuhlmann, Isabel J. Bauer, Eduardo Beltran, Lisa Ann Gerdes, Aleksandra Mezydlo, Amit Bar-Or, Brenda Banwell, Mohsen Khademi, Tomas Olsson, Reinhard Hohlfeld, Hans Lassmann, Tania Kümpfel, Naoto Kawakami, Edgar Meinl

**Affiliations:** 1 Institute of Clinical Neuroimmunology, Biomedical Center and University Hospitals, Ludwig-Maximilians-Universität München, Großhaderner Str. 9, 82152 Planegg-Martinsried, Germany; 2 Institute of Neuropathology, University Hospital Münster, Münster, Germany; 3 Therapeutic Immune Design, Department of Clinical Neuroscience, Karolinska Institutet, Stockholm, Sweden; 4 Institute for Diabetes and Obesity, Monoclonal Antibody Core Facility, Helmholtz Center Munich, German Research Center for Environmental Health (GmbH), Neuherberg, Germany; 5 Neuroproteomics, School of Medicine, Klinikum rechts der Isar, German Center for Neurodegenerative Diseases (DZNE), Technical University of Munich, Munich, Germany; 6 Research Unit Protein Science, Helmholtz Center Munich, Munich, Germany; 7 Department of Neurology, University of Pennsylvania, Philadelphia, USA; 8 Children’s Hospital of Philadelphia, Philadelphia, USA; 9 Neuroimmunology Unit, Department of Clinical Neuroscience, Karolinska Institutet, Stockholm, Sweden; 10 Center for Brain Research, Medical University, Vienna, Austria; 11 Munich Cluster for Systems Neurology (SyNergy), Munich, Germany

**Keywords:** Autoantigen, Multiple sclerosis, Neuroinflammation, Autoimmunity

## Abstract

Autoimmune disorders of the central nervous system (CNS) comprise a broad spectrum of clinical entities. The stratification of patients based on the recognized autoantigen is of great importance for therapy optimization and for concepts of pathogenicity, but for most of these patients, the actual target of their autoimmune response is unknown. Here we investigated oligodendrocyte myelin glycoprotein (OMGP) as autoimmune target, because OMGP is expressed specifically in the CNS and there on oligodendrocytes and neurons. Using a stringent cell-based assay, we detected autoantibodies to OMGP in serum of 8/352 patients with multiple sclerosis, 1/28 children with acute disseminated encephalomyelitis and unexpectedly, also in one patient with psychosis, but in none of 114 healthy controls. Since OMGP is GPI-anchored, we validated its recognition also in GPI-anchored form. The autoantibodies to OMGP were largely IgG1 with a contribution of IgG4, indicating cognate T cell help. We found high levels of soluble OMGP in human spinal fluid, presumably due to shedding of the GPI-linked OMGP. Analyzing the pathogenic relevance of autoimmunity to OMGP in an animal model, we found that OMGP-specific T cells induce a novel type of experimental autoimmune encephalomyelitis dominated by meningitis above the cortical convexities. This unusual localization may be directed by intrathecal uptake and presentation of OMGP by meningeal phagocytes. Together, OMGP-directed autoimmunity provides a new element of heterogeneity, helping to improve the stratification of patients for diagnostic and therapeutic purposes.

**Electronic supplementary material:**

The online version of this article (10.1186/s40478-020-01086-2) contains supplementary material, which is available to authorized users.

## Introduction

Inflammatory diseases of the CNS comprise a broad spectrum of disorders, multiple sclerosis is the most abundant one. A misguided immune response to autoantigens expressed in the CNS is expected to drive the disease in these patients [21, 51, 52, 68] and multiple targets of the autoimmune response have been suggested [8, 10, 15, 26, 28, 29, 32, 36, 42, 58, 62, 70]. The identification of autoantibodies to myelin oligodendrocyte glycoprotein (MOG) [53] and aquaporin-4 (AQP4) [39] in patients with clinical features similar to MS, have eventually resulted in the definition of separate diseases with important therapeutic consequences [44, 60, 61], but for most of the patients with inflammatory disorders of the CNS, the target of their autoimmune response has not been identified.

This study analyzes autoimmunity to oligodendrocyte myelin glycoprotein (OMGP), because this protein is specifically expressed in the CNS and there found on both oligodendrocytes and neurons. Therefore, OMGP could provide a target for both white and gray matter pathology. OMGP is a GPI-anchored protein and was originally identified as a 105 kDa glycoprotein of myelin in the CNS [48], which is also expressed by neurons [25]. The most studied function of OMGP is its role as a myelin derived inhibitor of axonal outgrowth [24], by binding to its receptors NgR [76] and PirB [4]. Although an autoimmune response against OMGP had been considered in studies looking at multiple CNS targets [13, 46], their abundance in patients has not yet been thoroughly determined and the pathogenic potential of Abs or T cells directed against OMGP was unknown. We set out to analyze autoantibodies targeting OMGP in patients classified in different disease entities. For the screening, we developed a live cell-based assay (CBA) with membrane anchored OMGP. Thereby, we found Abs to OMGP in 10/474 patients including 2.3% of patients with MS. Their anti-OMGP reactivity was confirmed with another cell-based system, where OMGP was displayed with its natural GPI anchor. To detect OMGP-specific T cells, we applied a recently developed sensitive method using bead-bound antigen as stimulant [8]. Further, we found that a soluble form of OMGP (sOMGP) is regularly present in the human cerebrospinal fluid (CSF) at high abundance in patients and controls. To gain further insight into the source and presence of OMGP in the CNS, we analyzed cultured oligodendrocytes and neurons from rodents and human oligodendrocytes derived from induced pluripotent stem cells (iPSCs) [17] and proved the presence of OMGP on these cells.

Having detected autoimmunity to OMGP in a subset of patients, we analyzed the pathogenic consequences of autoimmunity to OMGP in an animal model. To this end, we have established a transfer experimental autoimmune encephalomyelitis (EAE) model with OMGP-specific T cells. This yielded a novel type of EAE characterized by massive lymphocytic meningitis over the brain convexities. For analyzing the pathogenic potential of Abs against OMGP, we generated new monoclonal antibodies (mAbs) to OMGP in rodents. We found that anti-OMGP Abs, in contrast to anti-MOG mAbs, did not mediate demyelination or other tissue damage in the EAE animal model. These findings might be explained by the large amounts of sOMGP in the CSF: sOMGP might on the one hand block demyelination by anti-OMGP mAbs, while it is constitutively taken up and presented by meningeal phagocytes directing inflammation to the cortical convexities.

Together, this study detects autoimmunity to OMGP in a proportion of patients with CNS inflammation, shows that OMGP-specific T cells mediate a novel type of EAE and provides a mechanistic model for the lesion localization of OMGP-directed autoimmunity.

## Materials and methods

### Cloning of OMGP constructs

Two OMGP constructs were cloned for cell-based assays into the pEGFP-N1 vector. First, to display OMGP with a membrane anchor (OMGP-TM), the transmembrane spanning part of CD80 (P237-L306, UniProt Q549R2) was placed in between human OMGP (M1-S417, UniProt P23515) and EGFP. Second, to display OMGP with its natural GPI anchor (OMGP-GPI), we used a T2A ribosome-skipping element (GSGEGRGSLLTCGDVEENPGP), which allows to generate two proteins out of one mRNA [14]. To this end, the stop codon of EGFP was deleted in pEGFP-N1, followed by T2A and subsequent complete OMGP sequence, including GPI anchor signal peptide (V418-V440). Rat (UniProt, Q7TQ25) and mouse (UniProt, Q63912) OMGP were cloned similarly, while using the human GPI signal sequence. To obtain OMGP in soluble form, its C-terminal GPI signal sequence was replaced by a linker (GSGMGMGMGMM) plus Avi-tag sequence (GLNDIFEAQKIEWHE), which allows site-specific enzymatic biotinylation, followed by SGGSG linker and poly-His for IMAC purification, cloned into the pTT5 expression vector.

### Cell-based assays to detect antibodies to OMGP

OMGP-TM, OMGP-GPI (for human, mouse and rat OMGP), and pEGFP-N1 as a control were transiently transfected in HeLa cells. For screening, sera were diluted 1:50 and binding of IgG in serum was detected by FACS as described in detail in the Additional file 1.

### Production and purification of recombinant proteins

OMGP and Abs were produced in HEK293-EBNA cells by secretion of proteins into the supernatant. The recombinant mAb specific for OMGP (22H6-hIgG1) was produced with the same human IgG1 Fc-part as the anti-MOG specific mAb 8-18C5-hIgG1 [7, 66]. OMGP was enzymatically biotinylated via BirA ligase (Avidity, BirA500).

### Generation and characterization of new mAbs to OMGP

We have developed three new mAbs specific for OMGP, 22H6 (rat IgG2a/λ), 31A4 (mouse IgG2b/κ) and 14A9 (rat IgG2b/λ) as described in the Additional file 1. These mAbs show strong reactivity to human OMGP in cell-based assays, biotin-streptavidin ELISA and cross react to rodent OMGP. The variable region of 22H6 was cloned and expressed recombinantly with a human IgG1 Fc part, named 22H6-hIgG1.

### Detection of circulating antigen-specific B and T cells

To detect antigen-specific T cells a recently developed Fluorospot assay was applied [8], as described in the Additional file 1. To detect circulating OMGP-specific B cells, PBMCs were differentiated to Ab-secreting plasmablasts as described in the Additional file 1. For both assays cryopreserved PBMCs were used. The blood was withdrawn in EDTA tubes. PBMCs were isolated via Pancoll gradient, frozen in FCS with 10% DMSO in CoolCell™ freezing boxes at − 80 °C and then transferred to liquid nitrogen.

### ELISAs

Our ELISA detecting Abs to OMGP used recombinantly produced OMGP, which was enzymatically biotinylated on its Avi-tag. Further details and ELISAs to detect MOG-Ab h8-18C5 and to quantify C1q-binding of OMGP-specific Abs are in the Additional file 1. We developed an ELISA to measure soluble OMGP. To this end, we used our new anti-OMGP mAb 14A9 for coating, rat rIgG2b (BD Biosciences, 556968) was the control Ab. For detection, we used the polyclonal OMGP antibody (R&D, AF1674), which we had biotinylated with a biotinylation kit (abcam, ab201795). To detect OMGP in CSF, samples were diluted 1:30 in PBST-0.5% BSA.

### Western blot detecting soluble OMGP

CSF samples were pooled and approximately 30 × concentrated using filter columns (Amicon 3 kDa). These, together with recombinant OMGP and immunoglobulins were separated by SDS gel (Invitrogen, NP0321) and transferred on an activated (10% methanol) PVDF membrane (GE Healthcare, 10600023). As primary Ab, goat anti-OMGP (R&D, AF1674) was used. As secondary Ab, donkey anti-goat-IgG-HRP (Invitrogen, A16005) was used, because it shows only minimal cross-reactivity with human IgG due to absorption against human IgG. ECL prime solution (GE Healthcare, RPN2232) was used and signal was detected by digital imaging systems Odyssey Fc from Leica.

### Proteomic sample preparation and LC-MSMS measurement

20 µl of each CSF sample (n = 20) were analyzed by a combination of liquid chromatography with tandem mass spectrometry. Details are described in the Additional file 1.

### Culture and staining of primary cell cultures and spinal cord tissue

Hippocampi and cortices were taken from embryonic (E16) mice and oligodendrocyte precursor cells prepared from 6 to 9 days old mice. Spinal cord tissue was analyzed as free-floating 55 µm thick stainings. Details are described in the Additional file 1.

### Affinity purification of patient-derived Abs to OMGP

Plasma was precipitated by NH_4_SO_4_ reconstituted with PBS supplemented with 1/10 of volume of solubilization buffer (1.5 M NaCl, 60 mM Tris-base, 30 mM Tris–HCl, pH 7.4), which contains freshly added 1% Octyl-β-Glucopyranoside. After pre-absorption with a streptavidin column (GE Healthcare 17-5112-01), sample was applied to a streptavidin column loaded with 1 mg biotinylated OMGP, eluted with 0.1 M glycine, 0.15 M NaCl buffer (pH 3) and directly dropped into 500 µl of 1 M Tris solution (pH 8.8). Antibodies were concentrated with 50 K centrifugal filters (Amicon) and dialyzed against PBS.

### OMGP-specific T cell lines in the rat EAE model

To obtain antigen-specific T cell lines, Lewis rats were immunized with an emulsion of recombinant OMGP protein and complete Freund’s adjuvant basically as described previously [66]. This protocol yields preferentially CD4^+^ T cell lines. Flow cytometric characterization is described in Additional file 1.

Freshly restimulated T cells were injected intravenously for EAE induction (10 × 10^6^ OMGP-specific T cells/15 × 10^6^ OVA-specific T cells/1.1 × 10^6^ MBP-specific T cells). Clinical scores and weight of Lewis rats were checked daily. On day two after T cell transfer, animals were anesthetized by fentanyl/midazolam/medetomidine and 500 µg of antibodies were injected intrathecally into the cisterna magna as following: of the anti-OMGP mAbs (31A4, 14A9, 22H6, 22H6-hIgG1), respective isotype controls (mIgG2b, BD Pharmingen, 559530; rIgG2b BD Pharmingen, 556968; rIgG2a BD Pharmingen, 553926), 100 µg of anti-OMGP MAB1674 or control MAB005, 500 µg of 8-18C5-hIgG1 as well as HK3-hIgG1 neuroborreliosis control antibody [7] (produced in the same system) and 500 µg of human Igs (Kedrion). Three days later, rats were sacrificed and perfused with PBS and 4% PFA in PBS. Postfixation of dissected spinal cord and brain was carried out with 4% PFA in PBS at 4 °C.

### Histopathology of EAE rats

PFA-fixed brain and spinal cord tissue was dissected, embedded in paraffin and stained with hematoxylin/eosin, Luxol fast blue (LFB) myelin stain, and Bielschowsky silver impregnation. After antigen retrieval immunocytochemistry was performed. Details including the quantification are described in the Additional file 1.

### Phagocyte activation

IL-8 production of monocytic THP-1 cell line was analyzed upon stimulation with OMGP antigen (cell bound by HeLa cells) and the presence of OMGP antibody, leading to the formation of immune complexes. Details are described in the Additional file 1.

### Statistics

Prism 6 software from GraphPad was used for statistical analysis. For the identification of statistical differences, unpaired t-test, fishers exact test, Tukey’s honest significance test and two-way ANOVA were applied. The data is presented as mean ± standard error of the mean or standard deviation. *P*-values ≤ 0.05 were considered significant (* *p* ≤ 0.05; **p ≤ 0.01; ***p ≤ 0.001).

## Results

### Autoantibodies against OMGP in patients with CNS diseases

To identify patients with autoantibodies to OMGP, we developed two CBAs, OMGP displayed in the membrane with a transmembrane part (OMGP-TM) or with its physiological GPI anchor (Fig. S1). The quantification of the flow cytometer-based screening is explained in Fig. S1. We noted that in the OMGP-GPI assay, some transfected cells expressed only the upstream EGFP of the construct and do not display OMGP on their surface due to incomplete ribosome skipping (Fig. S1). Therefore, this assay was not used for screening, but for subsequent testing of patients’ Abs recognizing OMGP-TM.

**Fig. 1:**
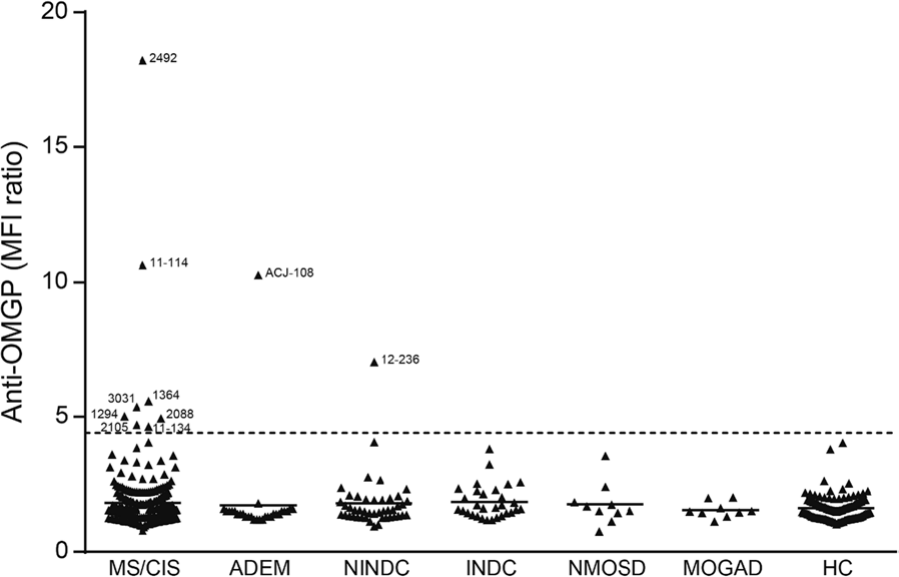
Identification of patients with Abs to OMGP. A total of 588 sera diluted 1:50 from seven cohorts were screened for OMGP autoantibodies using a cell-based assay (OMGP-TM) displaying OMGP with a transmembrane anchor. The following groups were analyzed: multiple sclerosis/clinical isolated syndrome (MS/CIS, n = 352), acute disseminated encephalomyelitis (ADEM, n = 28), non inflammatory neurological disease control (NINDC, n = 45), inflammatory neurological disease control (INDC, n = 30), neuromyelitis optica spectrum disorders (NMOSD, n = 10), MOG antibody-associated disease (MOGAD, n = 9) and healthy controls (HCs, n = 114). For the cut off evaluation, HCs were measured twice, except ten HCs samples coming from the Swedish cohort were analyzed once. The horizontal line at 4.4 represents the cut-off as mean plus 6 SDs of the HC cohort. From patients above the indicated threshold, the symbols show mean value of minimum two replicates. The numbers next to the symbols of the positive patients are the internal code numbers. Clinical details of these patients are in Table S2. ACJ-108 is a child with ADEM, patient 12–236 was diagnosed as psychosis, all other positive patients had MS. Index patient 2492 served as daily control and the value shown is the mean of 30 replicates. The raw data of the anti-OMGP reactivity of patient 2492 is shown in Fig. 2, of all other patients scored positive in Fig. S2

In total, 588 serum samples (Table S1) from 474 patients with different CNS diseases and 114 healthy controls (HC) were analyzed with OMGP-TM (Fig. 1). To avoid false-positive results, we set a stringent cut-off and calculated this as mean + 6 SDs of the HC group (Fig. 1). None of the healthy controls scored positive. Within the MS/CIS group, 8/353 (2.3%) showed autoantibodies to OMGP. When performing a group comparison, we did not detect a significant difference between this group and the HC cohort (two-tailed Fisher’s exact test, *p* = 0.208). One out of 28 pediatric patients (ACJ-108) diagnosed with acute disseminated encephalomyelitis (ADEM) had Abs to OMGP. When we screened sera from 45 non inflammatory neurological disease controls (NINDC), we noted that one patient diagnosed with psychosis (12-236) had Abs to OMGP (Fig. 1). In the inflammatory neurological disease control group (INDC), as well as neuromyelitis optica spectrum disorders (NMOSD) group and patients diagnosed with MOG antibody-associated disease (MOGAD), no OMGP autoantibodies are detected (Fig. 1). All ten OMGP^+^ patients were analyzed negatively for MOG IgG in our *in house* CBA [66] and in 4/10 patients AQP4 was also tested negatively throughout clinical routine. From six of the other anti-OMGP + patients no AQP4 data was available, since their clinical phenotype was clearly different from NMOSD.

**Fig. 2:**
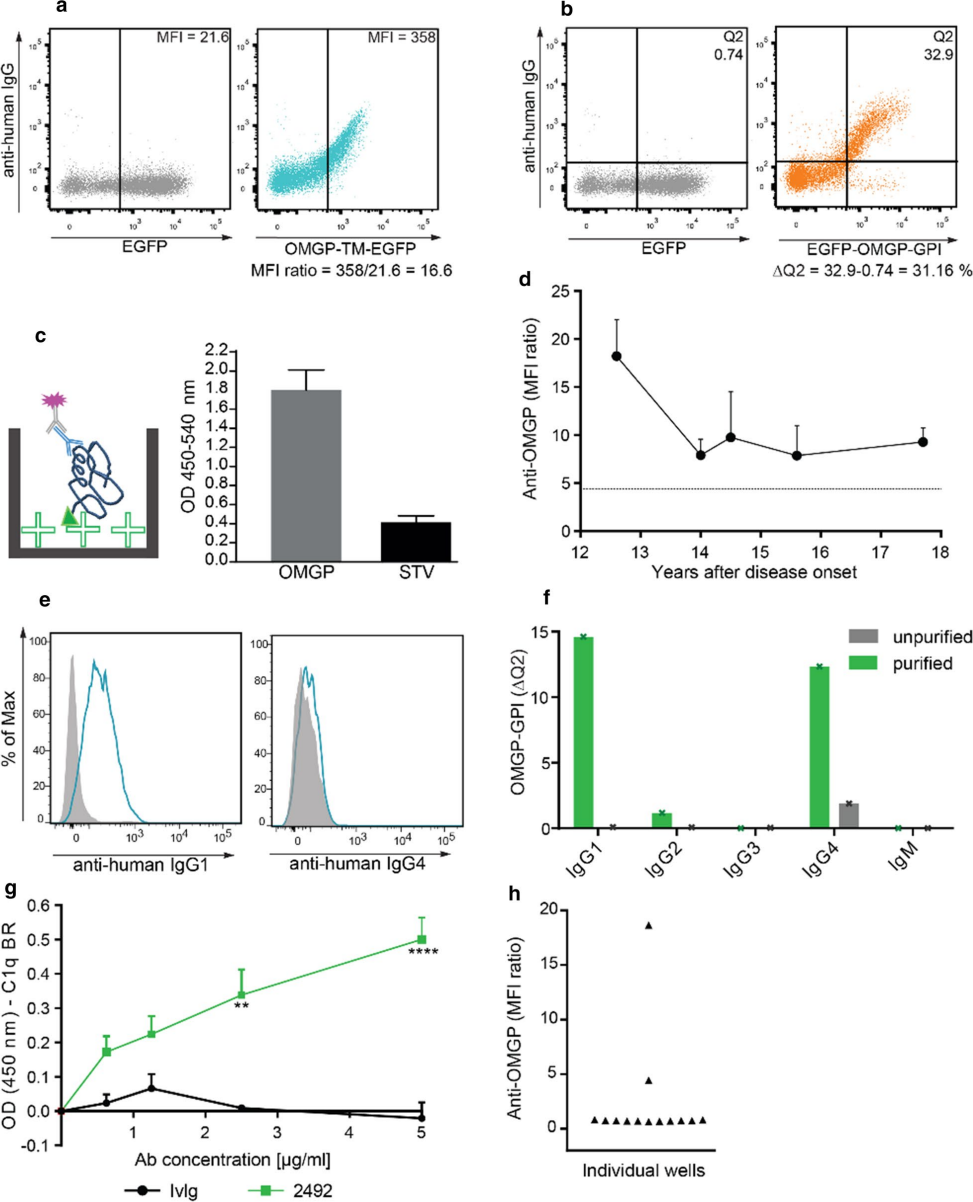
Features of Abs to OMGP in a highly reactive MS patient. **a** Reactivity of the serum (diluted 1:50) of patient 2492 in OMGP-TM CBA with a MFI ratio of 16.6. **b** Serum diluted 1:50 also recognized OMGP displayed with a GPI anchor. We calculated a ΔQ2 of 31.16%. **c** The Abs from this patient recognized OMGP also by ELISA. The serum (diluted 1: 100) was added to biotinylated OMGP bound to streptavidin (STV) plates and compared to STV plates alone. The bars represent the mean of 35 replicates with SD. **d** The longitudinal persistence of the anti-OMGP antibodies was analyzed with the OMGP-TM assay using serum samples from 12.6 to 15.6 years and plasma samples from 14, 14.5 and 17.7 years. Symbols represent at least means of two replicates, while first time point is measured 30x, second and last one 5x. Bars indicate SEM. Patient was treated with dimethyl fumarate during this period. **e** Isotype analysis of the OMGP-Abs in the OMGP-TM assay. The blue histogram displays IgG1 and IgG4 evaluation and grey the background of EGFP cells. **f** After OMGP autoantibody purification (Fig. S3), isotyping was reevaluated with the OMGP-GPI CBA. **g** C1q binding assay with patient’s derived purified OMGP autoantibodies using the STV-ELISA. Values represent mean of two experiments, bars indicate SEM. Two-way ANOVA with Bonferroni’s multiple comparison test is performed (*p* < 0.0001 ****, *p* < 0.01 **). **h** Identification of circulating OMGP-specific B cells. PBMC were differentiated to plasmablasts in 13 individual wells and then the anti-OMGP-reactivity was determined by OMGP-TM CBA

We tested the 10 sera that contained Abs to OMGP-TM also with OMGP-GPI and found that sera with a high response to OMGP-TM also showed a clear response to OMGP-GPI; the original FACS data of these 10 patients are shown in Fig. S2 and Fig. 2a, b. The clinical features of these patients are summarized in Table S2. The anti-OMGP response was IgG1 in all patients; some had in addition OMGP-specific IgG4 (Fig. 2 and Fig. S2).

We analyzed patient 2492 (index patient) with the highest response to OMGP in our cohort in detail (Fig. 2). This patient with RRMS for 18 years recognized OMGP-TM (Fig. 2a), OMGP-GPI (Fig. 2b) and OMGP by ELISA (Fig. 2c). We could analyze his autoantibodies to OMGP for the last 5 years and found that they persisted (Fig. 2d). The antibodies detectable in serum were of the IgG1 isotype (Fig. 2e). After affinity-purification of the OMGP-specific antibodies (Additional file 1, Fig. S3), we detected in addition to the IgG1 also OMGP-specific IgG4 (Fig. 2f). The affinity-purified Abs from this patient bound significantly more C1q than the control Abs (Fig. 2g). Furthermore, by differentiating circulating B cells in vitro to plasmablasts, we could also detect circulating OMGP-specific B cells in the blood of this patient (Fig. 2h).

### OMGP-specific T cells in patients with MS

To analyze the presence of OMGP-specific T cells in blood, we applied a recently developed highly sensitive assay for CD4^+^ T cells, in which the antigen is coupled to beads and multicolor FluoroSpot analysis of cytokine production by single cells is used as a read-out system [8]. We analyzed a new cohort of 28 MS patients (12 treated with natalizumab, since these patients might have enhanced numbers of autoreactive T cells in blood [8, 32] and 16 untreated) and 13 healthy controls (Table S3) by multicolor analysis with simultaneous detection of IFNγ, IL-17A, and IL-22. All three groups produced comparable levels of IFNγ and IL-17A upon anti-CD3 stimulation (Fig. S4A). Only for IL-22, the natalizumab group produced less. To quantify OMGP-specific T cells, ΔSFU were calculated by background subtraction of Avi-His coupled beads, as in a previous study using MOG-coupled beads [8]. For each cytokine a cut-off was calculated by the mean of HC values plus three SDs. Using these criteria, we found low levels of OMGP-specific T cells producing IFNγ, IL-22 and/or IL-17A in 1/12 natalizumab treated MS patients, 4/16 untreated MS patients, but in none of 13 healthy controls (Fig. S4B).

### High levels of a soluble form of OMGP is found in human CSF

In this study we generated new OMGP-specific mAbs, namely 14A9 (rat IgG2b), 31A4 (mouse IgG2b), 22H6 (rat IgG2a), 22H6-hIgG1, which were compared the commercially available MAB1674 (Fig. S5). These mAbs were analyzed in CBA (Fig. S5A), as well as OMGP-STV ELISA (Fig. S5B) and reacted comparable to MAB1674.

**Fig. 3:**
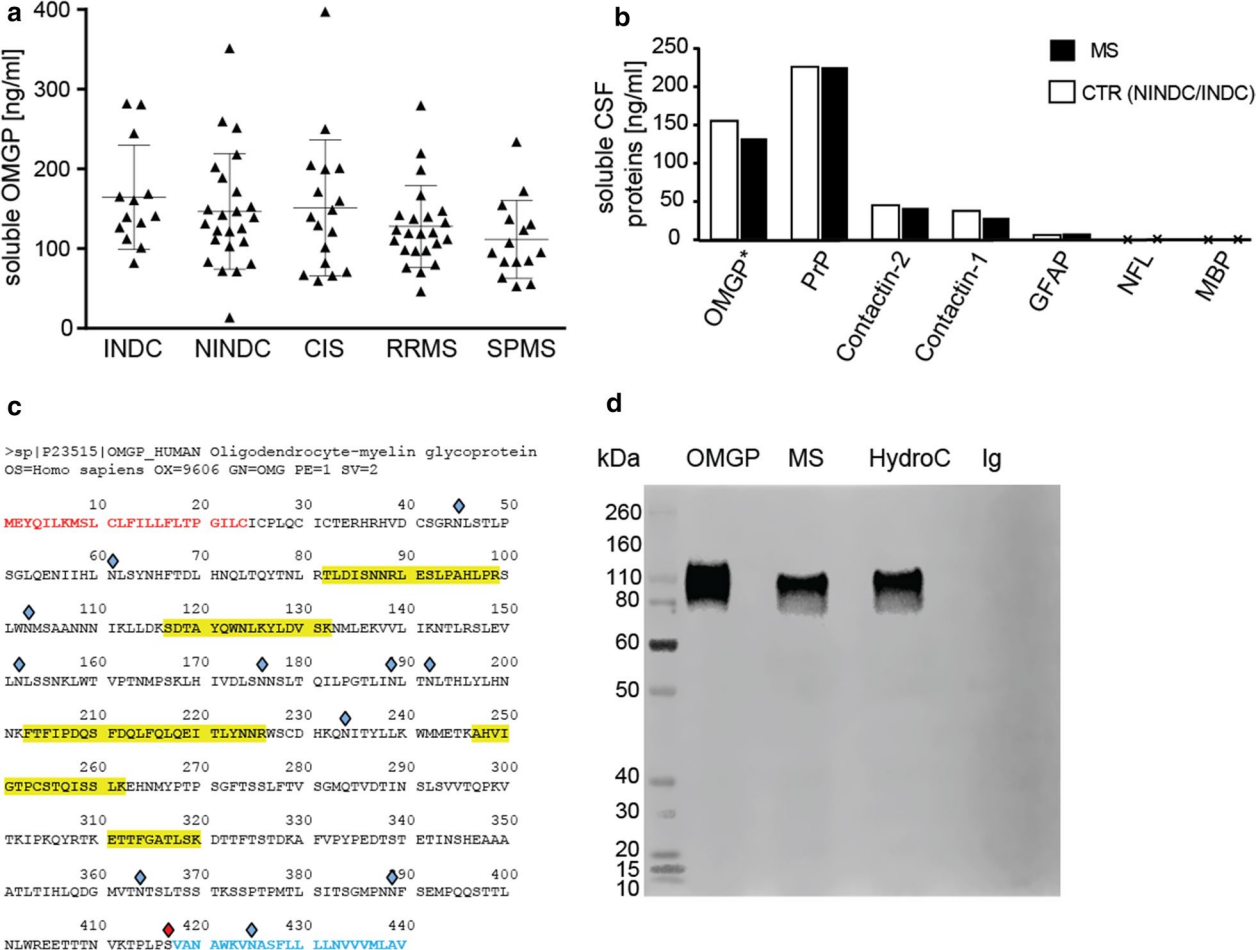
Soluble OMGP is abundant in the CSF. **a** Soluble OMGP was quantified in CSF from five cohorts of patients: inflammatory neurological disease control (INDC, n = 13), non inflammatory neurological disease control (NINDC, n = 24), clinically isolated syndrome (CIS, n = 17), relapsing–remitting MS (RRMS, n = 23) and secondary progressive MS (SPMS, n = 15). **b** Comparison of soluble OMGP to literature values of other soluble CSF proteins. Symbol (*) indicates that these data come from (**a**), displaying the mean of NINDC/INDC and RRMS cohorts, values for other proteins come from literature: PrP [47], Contactin and neurofilament light chain (NFL) [12], glial fibrillary acidic protein (GFAP) [1] and myelin basic protein (MBP) [33]. **c** Sequence of human OMGP with identified peptides in CSF by mass spectrometry (LC-MSMS). Peptides are identified with a peptide false-discovery rate < 1% and are marked in yellow. The signal peptide is shown in red, the GPI anchor sequence in blue. Sites of glycosylation (according to Uniprot) are marked with blue diamonds; the GPI anchor site is marked with a red diamond. **d** Recombinant human OMGP (50 ng), pooled and 30-fold concentrated CSF from patients with MS or normal pressure hydrocephalus (HydrC), and 30 µg of human Ig (Ig) were loaded and separated by SDS-PAGE (full undigested gel), blotted and detected with polyclonal OMGP Ab (AF1674)

To evaluate the presence of a soluble form of OMGP (sOMGP), we have developed an ELISA, using one of our new mAbs (14A9) and a commercially available polyclonal Ab for detection (Fig. S6). We found that sOMGP was abundantly present in each of the analyzed 92 CSF samples (Table S4) with an overall mean of 151 ng/ml. Samples from INDCs, NINDCs as well as CIS, RRMS and SPMS patients had similar levels of sOMGP in the CSF (Fig. 3a). We analyzed, if the levels of sOMGP in the CSF were related to acute inflammation. To this end, we have compared six samples from CIS patients during active disease with 11 samples of CIS patients during remission and noted no difference. Also, the sOMGP levels of 8 RRMS patients obtained during relapse with the samples from 16 RRMS patients taken during remission did not show a difference. To appreciate the high level of sOMGP, we included RRMS and control values for comparison to other CNS proteins previously measured in other studies (Fig. 3b). This shows that PrP is present in a similar, slightly higher concentration [47] and two other GPI-anchored proteins, contactin-1 and contactin-2 were about 3–5 times lower [12] than sOMGP. Levels of GFAP [1], MBP and NFL [12, 33] were more than 10–100 fold lower than the level of sOMGP in the CSF. We found no correlation between the sOMGP concentration and the age of patients.

We went on to analyze the sOMGP in the CSF in more detail. By mass spectrometry, we detected five peptides of OMGP (Fig. 3c). Western blot of CSF of patients with MS or hydrocephalus revealed a molecular weight of about 105 kDa, the full length of OMGP (Fig. 3d).

### Expression of OMGP by neurons, immature and mature oligodendrocytes

**Fig. 4:**
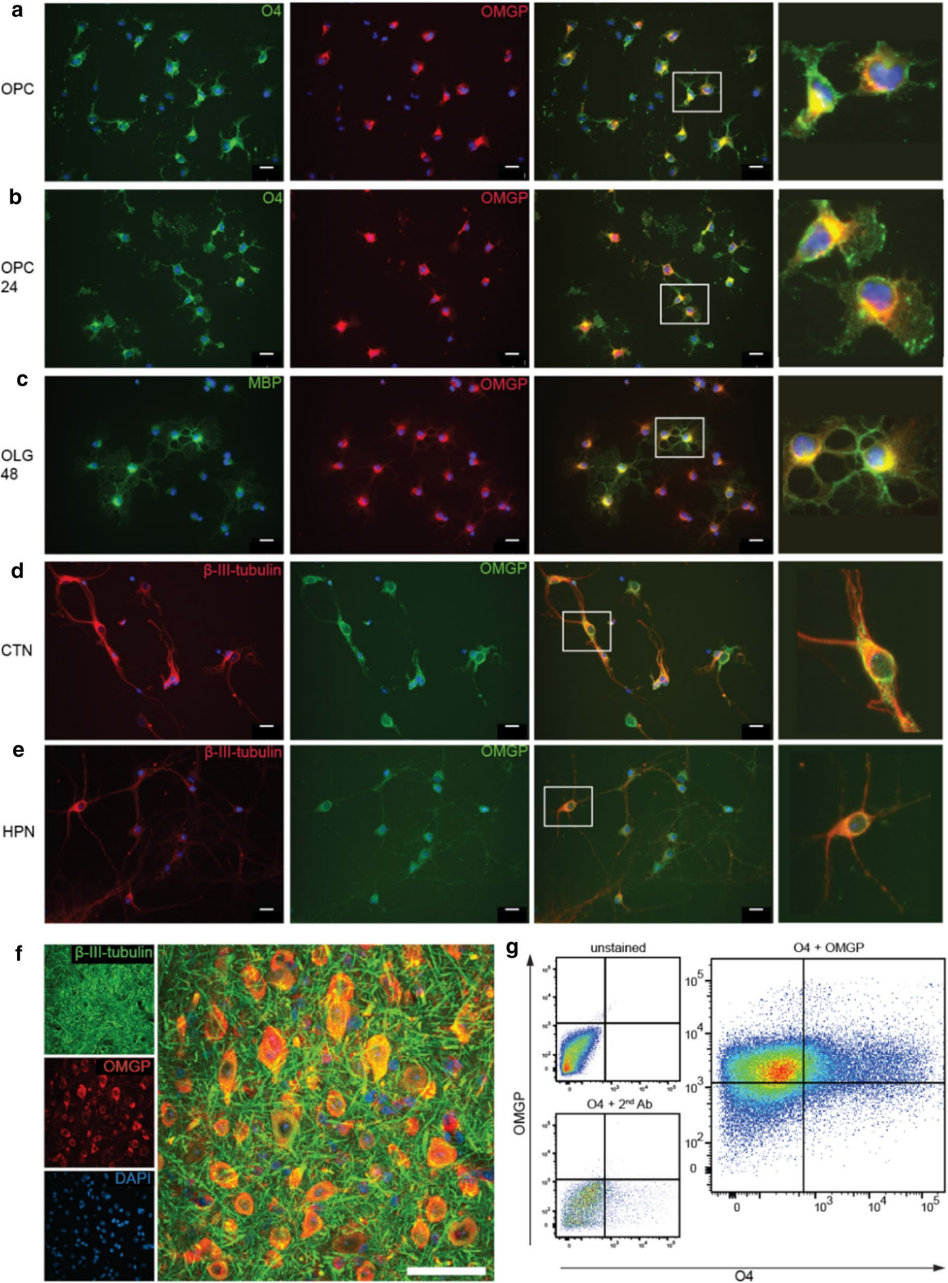
OMGP is displayed by neurons, immature and mature oligodendrocytes. Primary mouse oligodendrocyte precursor cells (OPC) (**a**) were differentiated for 24 h (**b**) or 48 h (**c**). A double staining was performed for OMGP (22H6) with the early oligodendroglial marker O4 (**a**, **b**), or MBP indicating differentiated oligodendrocytes (**c**). **d** Mouse cortical neurons (CTN) as well as (**e**) hippocampal neurons (HPN) were double-stained with the neuron-marker β-III-tubulin and OMGP (22H6). (**a**–**e**) scale bars represent 20 µm and white rectangles mark the zoomed area. (**F**) Spinal cord tissue sections of 55 μm were stained with anti-OMGP (22H6, red) and β-III-tubulin (green) for visualization in grey matter. Images are stacks from confocal microscopy with 60x magnification and white scale bar indicates 50 μm. **g** Human oligodendrocytes were double-stained with anti-OMGP (22H6-hIgG1) and O4. Quadrants were set with human IgG and secondary Abs as control

Using primary cultures from mice, we saw OMGP on O4^+^ immature oligodendrocytes and mature MBP^+^ oligodendrocytes with a branching morphology (Fig. 4a–c). All three mAbs 31A4, 14A9 and 22H6 were used for staining and gave a similar pattern, 22H6 is displayed as representative example. We detected OMGP also in cortical (Fig. 4d) and hippocampal neurons (Fig. 4e). β-III-tubulin was used as neuronal marker. OMGP was detected surrounding neuronal somata as well as in neurites (Fig. 4d, e). In situ, we stained OMGP in spinal cord neurons (Fig. 4f). Finally, we analyzed the expression of OMGP in human oligodendrocytes obtained through differentiation of iPSC [17]. OMGP was detected on O4^+^ and O4^−^ cells in these oligodendrocyte cultures (Fig. 4g). This part of our work confirms previous studies in rodents [11, 25, 30, 48] and extends it to human oligodendrocytes.

### Massive meningitis above the cortical convexities induced by OMGP-specific T cells

**Fig. 5:**
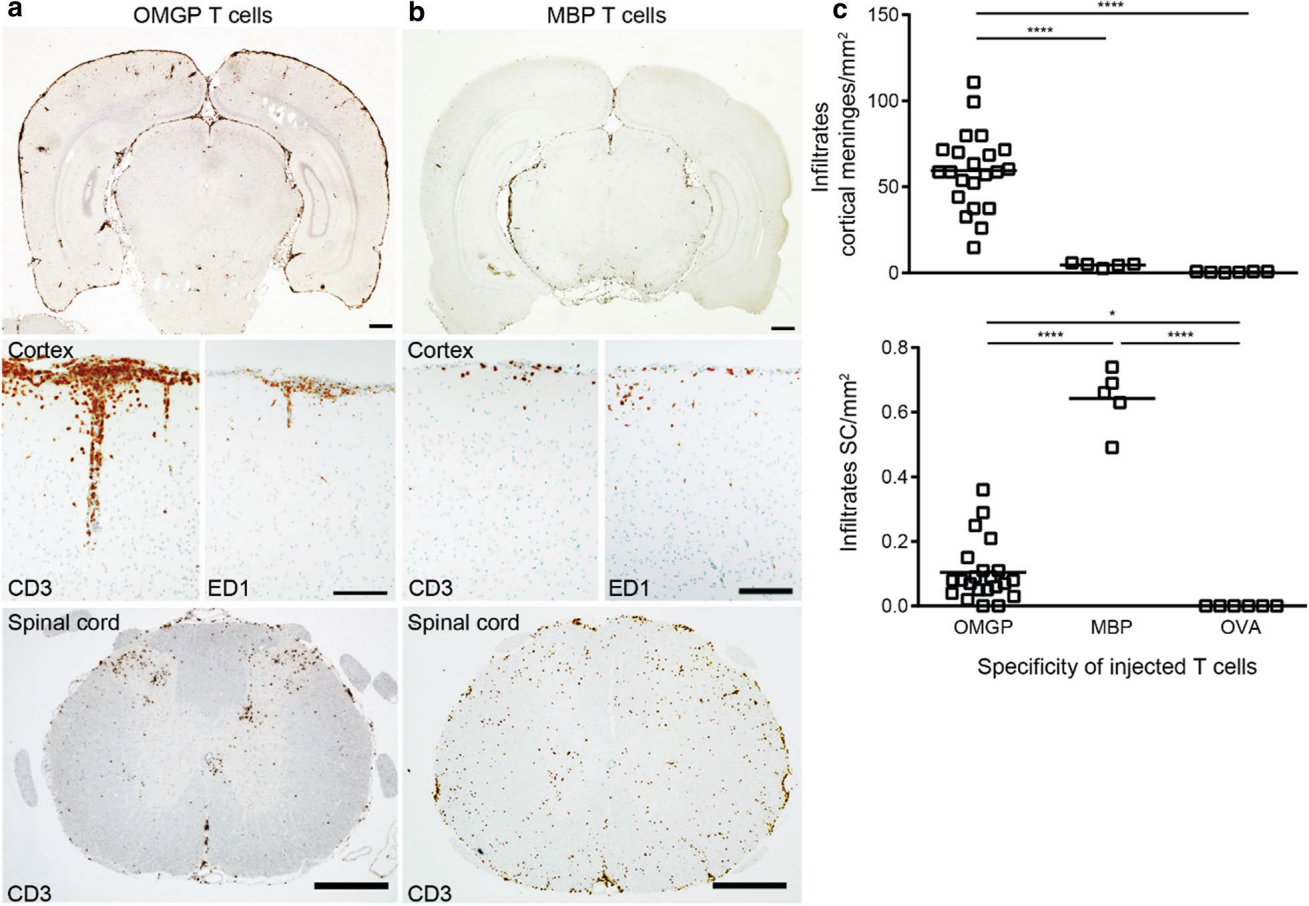
OMGP-specific T cells mediate cortical meningitis. Lewis rats were intravenously injected with OMGP-specific T cells (**a**) or MBP-specific T cells (**b**) and sacrificed after 5 days. Tissue sections were stained with mAbs to CD3 (T cells) or ED1 (macrophages), developed with DAB as substrate resulting in a brown color. Nuclei were counterstained blue. **a** OMGP-specific T cells mediate a cortical meningitis (images display representative histology seen with OMGP-specific T cells). Also an infiltration of the spinal cord is seen, preferentially in the dorsal horn (image from an animal with a high number of infiltrates in the spinal cord was selected to show the preferential localization of the infiltrates in the dorsal horn). **b** After transfer of MBP-specific T cells, lower number of CD3^+^ T cells in the cortex and few ED1^+^ macrophages are found in the cortical meninges. In contrast, a prominent infiltration of the spinal cord is seen. **a**, **b** Scale bars in the upper row represent 1 mm, in the middle row 0.1 mm and in the lower row 0.5 mm. **c** Quantification of the infiltrates by hematoxylin/eosin staining. The values are given as infiltrates per mm^2^. Cortical meninges: each dot represents the mean of the infiltrates of one animal, where 6 microscopic fields were counted. Spinal cord (SC) quantification: each dot represents the counted average of 20 SC cross sections. The 22 animals injected with OMGP-specific T cells include animals without an additional injected antibody, with control Abs and with OMGP Abs (data were pooled, because these three subgroups with OMGP-specific T cells were similar Fig. S7A). Tukey’s honest significance test is performed (*p* < 0.0001 ****, *p* < 0.05 *)

The transfer of OMGP-specific T cells into Lewis rats induced an inflammation in the CNS with an unusual distribution of the infiltrates (Fig. 5a). There was massive meningitis over the cerebral cortex, but little inflammation in the meninges covering the cerebellum. OMGP-specific T cells induced meningitis with some spread into the Virchow-Robin space of large cortical vessels. The inflammatory reaction was largely present in three areas of the CNS, namely the cortex, the medulla oblongata (next to the fourth ventricle) and in the gray matter in the spinal cord (Fig. 5a).

In the spinal cord, the inflammation was most prominent in the gray matter of the dorsal horn and around the central canal. The inflammation was largely composed of T cells with little contribution of ED1^+^ (CD68^+^) macrophages (Fig. 5a). During this study, we observed this histological pattern in a total of 22 rats comprising 3 rats that received only OMGP-specific T cells alone, 11 rats that received in addition control Igs and 8 rats received OMGP Abs (Table S5 displays animals used in this study). All the animals with OMGP-specific T cells alone, plus control Abs or OMGP Abs, did not show any difference (Fig. S7A). The typical EAE scores, which largely reflect paresis due to spinal cord lesions but do not indicate cortical deficits, were zero.

A very different pattern of inflammation was induced by MBP-specific T cells (Fig. 5b). Here, hardly any inflammation was seen in the cortex, but massive in the spinal cord, cerebellum and medulla oblongata. The infiltrates were located perivascular and in the parenchyma, and were associated with a large recruitment of macrophages. Our quantitative analysis of the histological stainings elaborate the difference between the pathology mediated by MBP- and OMGP-specific T cells (Fig. 5c): OMGP-specific T cells induce a significantly stronger inflammation in the meninges over the cortical convexities, while MBP-specific T cells induce a significantly stronger inflammation in the spinal cord.

As a further control we used T cells specific for OVA (6 rats) which were included in the histological quantification (Fig. 5c). These T cells did not induce inflammation, neither the T cells alone (n = 3) nor with control antibodies (IvIg, n = 3) or with the humanized anti-MOG mAb 8-18C5-hIgG1, as it was shown in our previous study [66].

Having seen that MBP-specific and OMGP-specific T cells mediate a different pathology, we analyzed if this could be linked to different cytokine profile or surface markers. We found that OMGP-, MBP-, and also OVA-specific T cells express the same surface proteins (Fig. S7B): CD4^+^, αβTCR^+^, activation markers CD25^+^ and CD134^+^, as well as adhesion molecules CD44^+^, CD11a/b^+^, CD49d^+^ and are negative for naïve markers CD45RA^−^/CD45RC^−^. Additionally, these three T cell lines share the same cytokine profile (Fig. S7C): They were largely Th1 cells with a strong IFNγ production with a little contribution of IL17. Arguing that the different pathology induced by MBP- and OMGP-specific T cells is due to their antigen-specificity, since these T cell lines express similar surface and activation markers.

### Priming for anti-MOG mediated demyelination by OMGP-specific T cells

**Fig. 6:**
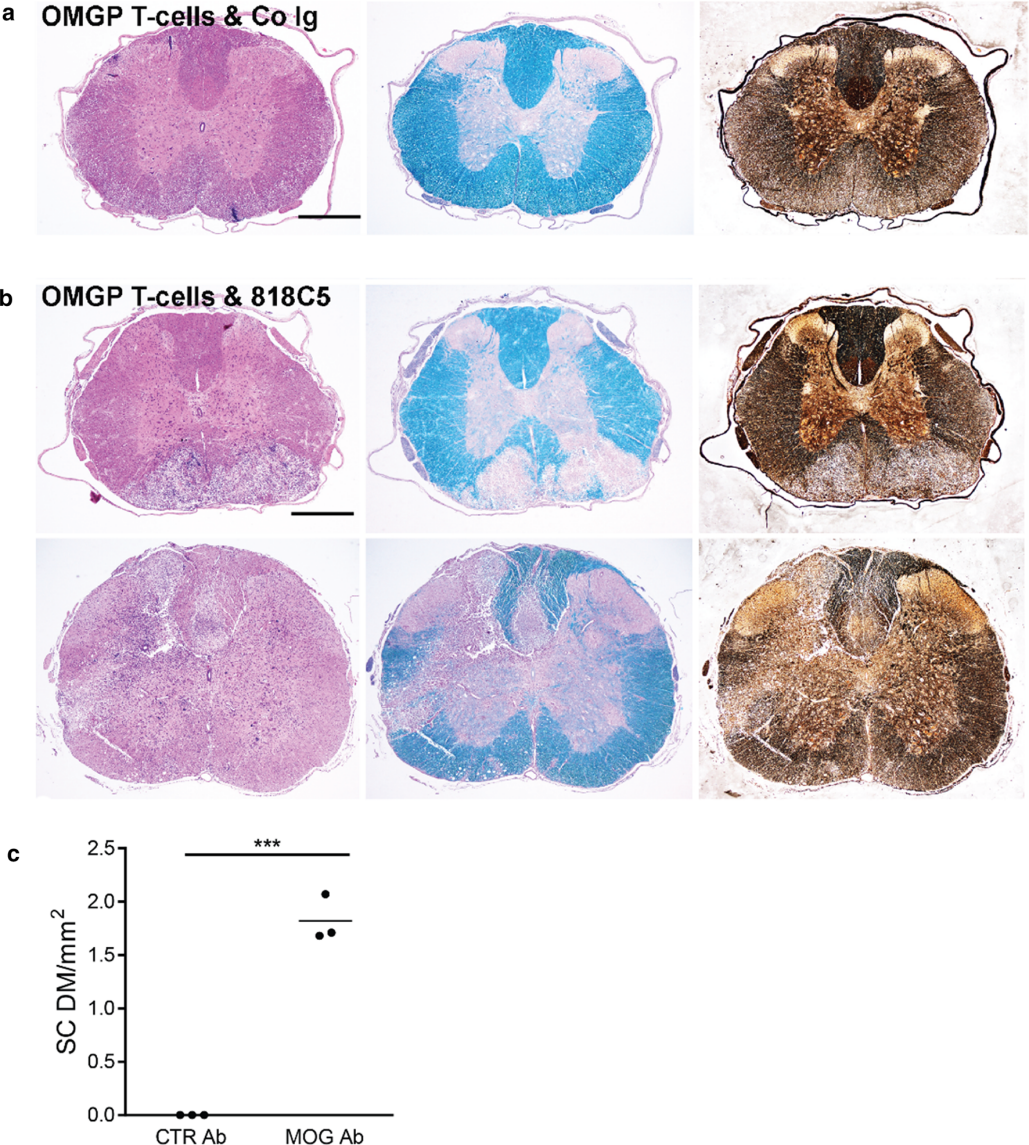
OMGP-specific T cells pave the way for anti-MOG mediated demyelination in gray and white matter of the spinal cord. Lewis rats were injected with OMGP-specific T cells, 2 days later either a control Ig (IvIg) (**a**) or the MOG-Ab 8-18C5-hIgG1 (**b**) was given and after 3 more days, the animals were sacrificed. Cross sections of the spinal cord were stained with H&E (left), LFB (middle), or Bielschowsky’s Silver Staining (right). **b** T cells injected together with MOG antibody (8-18C5-hIgG1), demyelination and neuronal destruction shown by Bielschowsky’s staining. Large demyelinating areas are seen in the white matter (upper part of **b**) and in white plus gray matter (lower part of **b**) along with axonal injury. Scale bar represents 1 mm. **c** Spinal cord (SC) demyelination (DM) was quantified from the LFB staining. The area of DM is shown in mm^2^ per SC section. (*p* < 0.001 ***; t-test)

We tested whether OMGP-specific T cells breach the blood–brain barrier and synergize with MOG-specific Abs to mediate demyelination. To this end, six animals received systemically OMGP-specific T cells. Two days later, three of them received intrathecally the humanized MOG-specific mAb 8-18C5-hIgG1 and three human control immunoglobulin (IvIg). The co-transfer of 8-18C5-hIgG1 induced slight clinical symptoms (mean score 0.3 at day 5 of 3 animals), while the control animals with the IvIg did not get sick. OMGP-specific T cells with control Abs induced inflammation, but no demyelination (Fig. 6a), while OMGP-specific T cells plus anti-MOG resulted in demyelination (Fig. 6b). The demyelinating lesions were found either in the white matter (Fig. 6b, upper row) or comprised both white and gray matter (Fig. 6b, lower row). In the spinal cord the lesions were perivascular as well as subpial, related to perivascular or meningeal inflammatory infiltrates. As shown before, subpial demyelination in a MOG antibody driven pathogenesis is in general not associated with major damage of the glia limitans, since the glia limitans is not a diffusion barrier for proteins, such as antibodies or complement from the CSF into the brain. However, the glia limitans may be infiltrated by immune cells in some of the lesions. The quantification of the spinal cord demyelination is displayed in Fig. 6c. As reported previously, no demyelination was observed when the mAb 8-18C5-hIgG1 was given together with OVA-specific T cells [66].

OMGP-specific T cells plus MOG-Ab 8-18C5-hIgG1 induced also demyelinating lesions in the cortex (Fig. S8A), whereas the human control Igs did not (Fig. S8B). These lesions showed a massive inflammation (H&E, CD3), with little activation of macrophages (ED1), but massive Iba1 activation (phenotypically microglia). There was a loss of CNP (2’,3’-cyclic nucleotide 3’ phosphodiesterase) and MOG positive myelin, without a loss of oligodendrocytes. Within these lesions, there was a massive deposition of the injected human Ig and of C9neo along with rat IgG, reflecting the breached blood–brain barrier. We also noted a partial loss of AQP4; this is a consequence of gliosis, since under inflammatory conditions, protoplasmic astrocytes are enlarged, which express a lot of GFAP and partially lose their extensions, where AQP4 is mostly localized [63]. Further, there was a subpial decoration of myelin with the injected MOG-specific human IgG (Fig. S8). There was very little or no subpial demyelination, but the demyelination was mainly seen in perivascular spaces around larger cortical vessels. The reason for this observation is not entirely clear, but may be related to the fact that macrophage infiltration and complement activation mainly occurred around inflamed vessels (Fig. S4). It is likely that inflammatory cells and mediators, such as complement, reach higher concentrations around inflamed parenchymal vessels, while when present in the subarachnoid space, they are diluted and washed away by CSF and CSF flow.

We also tested by MOG ELISA the presence of the injected 8-18C5-hIgG1 in the serum of the rats when they were sacrificed: All sera from the three animals injected with the 8-18C5-hIgG1 were highly positive for MOG Abs in the peripheral blood, whereas the rats injected with control antibodies did not show a signal above background. Thus, the intrathecally injected IgG was detected in serum and could mediate demyelination by entering areas with disturbed blood–brain barrier from the blood stream explaining the perivascular IgG deposition seen in Fig. S8A. In addition, the MOG-Abs may also diffuse along the perivenous spaces from the subarachnoid space into the tissue.

### No demyelination, but activation of phagocytes mediated by Abs to OMGP

We tested, whether anti-OMGP-Abs mediate demyelination as we observed for MOG-Abs. We applied five different OMGP-specific mAbs, namely 14A9 (rat IgG2b), 31A4 (mouse IgG2b), 22H6 (rat IgG2a), 22H6-hIgG1, which were generated in this study and the commercially available MAB1674. These mAbs cross-react with rodent OMGP as seen by cell-based assay, also recognized OMGP by ELISA, had different isotypes and accordingly different binding to C1q (Fig. S5). We injected 2 days after the transfer of encephalitogenic MBP-specific T cells, 500 µg of the OMGP-mAbs 14A9, 31A4, 22H6, or isotype specific control Abs intrathecally (Table S5). The animals were sacrificed 3 days later and analyzed histologically. None of these antibodies induced demyelination under these conditions. For comparison, 8-18C5-hIgG1 was used, which induced a strong demyelination in the context of MBP-specific T cells, as seen in a previous study [66].

Since we found that OMGP-specific T cells open the blood–brain barrier and paved the way for anti-MOG mediated demyelination (Fig. 6 and Fig. S8), we also injected OMGP-specific mAbs together with OMGP-specific T cells. We used MAB1674 and our new mAb 22H6 (rIgG2a). None of these Abs induced demyelination, whereas 8-18C5-hIgG1 induced a strong demyelination as seen before (Fig. 6). Since the 8-18C5-hIgG1 had a human IgG1 backbone, which strongly activates complement, we cloned the mAb 22H6 in the same vector and produced it recombinantly, 22H6-hIgG1. Indeed, this enhanced the C1q binding to 22H6-hIgG1 mAb (Fig. S5C), but injection along with OMGP-specific T cells did not induce demyelination. Together, all these experiments indicate that Abs to OMGP did not mediate demyelination, in contrast to the anti-MOG-mAb 8-18C5-hIgG1.

Since certain mAbs enhance immune cell activation after binding to their antigen [20, 66], presumably by binding of immune-complexes to FcR [35], we tested in vitro the activation of phagocytes by an OMGP mAb in the presence of its antigen. For these experiments, we used 22H6-hIgG1 and the human phagocyte cell line THP-1. We observed that this OMGP-specific Ab activated these phagocytes in the presence of its antigen as seen by induced secretion of IL-8 (Fig. S9).

## Discussion

We report here that (1) autoimmunity to OMGP can be detected in a proportion of patients with inflammatory diseases of the CNS, (2) high levels of sOMGP are constitutively present in the CNS and (3) OMGP-specific T cells mediate a novel type of EAE with inflammation in the meninges of the cortical convexities.

To analyze the presence of autoimmunity to OMGP in patients with inflammatory CNS diseases, we have established a live cell-based assay, since autoantibodies against membrane-anchored proteins are in many instances reliably detected with cell-based assays [38, 60]. We used a stringent cut-off to exclude potential false positive ones in this first report of OMGP autoimmunity. We found that 8/353 (2.3%) MS patients, 1/28 (3.6%) of children with ADEM have autoantibodies against OMGP, but none of the healthy donors. Since OMGP is GPI-anchored, we confirmed recognition of OMGP by displaying OMGP in its GPI-linked form. We noted that also one patient diagnosed with psychosis showed a clear autoantibody response to OMGP. Our observations that autoimmunity to OMGP induces cortical pathology in an animal model, might inspire larger studies to learn whether there is a subset of psychosis patients with auto-Abs to OMGP. In children with ADEM, around 20% of the patients have Abs to MOG [53]. Our observation that 1/28 children with ADEM has auto-Abs to OMGP is compatible with the view that different autoantigens are targeted in these patients. We noted that the 10 patients with Abs to OMGP we identified did not show a striking common phenotype. This is similar to experiences with anti-MOG and anti-GAD65. Abs to MOG are found in patients with different clinical phenotypes such as childhood ADEM with encephalopathy [46, 53], isolated optic neuritis [59], NMOSD [44, 60, 81], rarely in patients fulfilling the criteria of MS [65] and in cortical encephalitis with epilepsy [54]. A consensus is emerging that despite these clinically different features, patients with MOG-Abs should be grouped as a separate disease [44, 81]. Also, patients with anti-GAD65 show different neurological syndromes like stiff person syndrome, cerebellar ataxia, limbic encephalitis, epilepsy, or oculomotor dysfunction [38, 71]. The different localizations of OMGP and our animal model, showing that autoimmunity to OMGP can result in cortical encephalitis/meningitis, lesions in the spinal cord and may pave the way for demyelination by antibodies of a different specificity, might explain why different clinical features can be associated with autoimmunity to OMGP. Further samples should be analyzed to establish the spectrum of syndromes associated with autoimmunity to OMGP.

The isotype of anti-OMGP-Abs was largely IgG1, similarly as anti-MOG or anti-AQP4. In some patients we noted in addition to the IgG1 also an IgG4 response. The IgG4 contribution to OMGP-specific Abs we could formally prove with affinity-purified Abs from a highly reactive MS patient. IgG4 is at the end of the possible IgG switch-chain in humans (IgG3 → IgG1 → IgG2 → IgG4) and typically indicates repeated antigen exposure [75]. A co-occurrence of IgG1 and IgG4 has also been observed in patients with CASPR2-specific Abs [74]. Since isotype switching to IgG1 and IgG4 is typically the result of a germinal center reaction involving cognate T cell help, this indicates the presence of OMGP-specific T cells in these patients.

We set out to get a first insight into OMGP-specific T cells using a recently developed highly sensitive technology to detect CD4^+^ T cells, which applies bead-coupled antigens and uses a multicolor FluoroSpot as read-out [8]. Thereby, we could detect OMGP-specific T cells in MS patients secreting IFNγ, IL-22 or IL-17A at low frequency. This low frequency of autoreactive T cells is consistent with previous experiences with MS and other human autoimmune diseases [28, 29]. For technical reasons the identification of patients with an antigen-specific autoimmune response is typically done by measuring autoantibodies (e.g. against MOG or AQP4) rather than quantifying the low frequency autoantigen-specific T cells. In contrast to autoimmune diseases, in certain infectious diseases like tuberculosis, the measurement of the high frequency of microbe-specific T cells is of diagnostic relevance [50]. The future application of further technologies such as libraries of amplified T cells [10, 22], peptide libraries [32] and tetramers detecting CD8^+^ T cells [62] will give a deeper insight into features of OMGP-specific T cells. In addition, antigen-specific tolerance is a promising specific future therapy [43, 69]. OMGP is a novel candidate antigen to be included in future cocktails for antigen-specific therapy.

Further, we found that a soluble form of OMGP is constitutively present in enormously high amounts in the CSF. sOMGP was recently detected in the supernatant of neuronal and even more in oligodendrocyte cultures [72]. The levels of sOMGP we measured in the CSF are about 10–100-fold higher than previously reported for MBP [33], GFAP [1] or neurofilament light chain [12]. We found that the levels of sOMGP were not further elevated during a relapse; this is presumably due the enormously high basal level of sOMGP, which is orders of magnitude higher than that of MBP; therefore increased levels of MBP are seen in active disease, but not of OMGP. The presence of sOMGP in human [16] and murine [72] CSF was noted in previous proteomic studies. A similar abundance in the CSF as we observed for OMGP has been reported for PrP, contactin-1, and contacin-2 [47, 73]. All of these proteins are GPI-anchored. OMGP was previously found to be released by exogenously added phosphatidylinositol-specific phospholipase C (PI-PLC) [48, 76]. GPI-linked proteins can principally also be shed by ADAM proteases as has been worked out for PrP [40]. The size of sOMGP we found in the CSF (105 kDa) is basically full-length and compatible with cleavage of OMGP by a lipase or by an ADAM protease close to the membrane, but the biochemical details of the shedding of OMGP have yet to be elaborated.

We tested the pathogenic potential of OMGP-specific T cells and mAbs to OMGP in an animal model in the Lewis rat. Strikingly, OMGP-specific T cells induce a novel type of EAE characterized by infiltrations in meninges around the cortical convexities. This localization is very different from the localization mediated by MBP-specific T cells [6]. We assume that this unusual localization of the inflammatory lesions is due to the enormous levels of sOMGP in the spinal fluid. From there it is taken up by meningeal macrophages and presented to OMGP-specific T cells. We suppose that sOMGP in the CSF is derived from both neurons and oligodendrocytes. We saw OMGP in rodent O4^+^ oligodendrocyte progenitor cells and MBP^+^ mature oligodendrocytes, consistent with the localization of OMGP in myelin [11] and oligodendrocytes [25, 30]. We extend this by showing that also human iPSC-derived oligodendrocytes (both O4^+^ and O4^−^ cells) express OMGP. We also detected OMGP in cultured neurons, which is consistent with the immunohistochemical localization of OMGP in neurons of the dorsal horn of the spinal cord and of the hippocampus [25]. We propose that sOMGP is transported to the perivascular space and the CSF by interstitial flow [31, 67] and then taken up by perivascular and meningeal macrophages [3, 19, 34]. In the presence of OMGP-specific T cells, this results in cortical meningitis. It has been shown that leptomeningeal macrophages present local myelin to encephalitogenic T cells [5, 37].

The second pathological feature of autoimmunity to OMGP, inflammation in the gray matter of the spinal cord, might be based on neuronal expression of OMGP. In inflammatory diseases of the CNS, precise localization of the lesions is determined by the site of antigen expression: For example, T cells specific for MBP induce inflammation largely in the white matter of the lumbar spinal cord [6] while T cells against contactin-2 [15] and β-synuclein [42], which are found in gray matter, target the inflammatory response to the gray matter. We found OMGP in spinal cord neurons in situ, which might be shed and subsequently presented by adjacent microglia to OMGP-specific T cells.

The third feature of OMGP-specific T cells is opening of the blood–brain barrier and paving the way for demyelination by MOG-Abs. Similar synergy of encephalitogenic T cells and MOG-Abs has been observed in several models [15, 23, 41, 66]. Together with OMGP-specific T cells, we observed a strong perivenous confluent demyelination, associated with Ig-deposition and complement activation. The anti-MOG Ab induces this demyelination most likely by entering from the periphery although we had applied the Ab intrathecally, since we observed high levels of anti-MOG reactivity also in the serum after intrathecal application.

Since evidence has been provided that OMGP is expressed on the outside of myelin [11] like MOG [60], we addressed the question whether antibodies against OMGP would mediate demyelination as antibodies to MOG [66]. We have tested four different mAbs specific for OMGP for inducing demyelination in combination with T cells specific for MBP or OMGP that breach the blood–brain barrier. These anti-OMGP mAbs had different isotypes and included also complement activating isotypes seen by C1q binding. None of the anti-OMGP mAbs induced demyelination. In order to exclude the possibility that all of our anti-OMGP mAbs just lack the appropriate Fc part to activate the effector mechanisms mediating demyelination, we cloned the Ig chains from one of them and produced the Ab recombinantly with the same human IgG1-Fc part as our recombinant MOG-Ab 8-18C5-hIgG1, which we used as a positive control [66]. The recombinant OMGP-Ab with a human IgG1 had an enhanced C1q binding, but also this mAb did not mediate demyelination. Together, all our experiments argue that anti-OMGP mAbs do not mediate demyelination in contrast to anti-MOG mAbs. The high level of sOMGP in the CSF (and presumably also in the brain parenchyma) might absorb the anti-OMGP Abs thus preventing Ab-mediated demyelination. Also, shedding of OMGP might be induced by Ab-binding. Alternatively, we cannot exclude that the affinity to rat OMGP of the mAbs we tested was too low, although we detected a cross-reactivity to rodent OMGP. Autoantibodies can be pathogenic by different mechanisms including complement activation [64] and enhancing activation of cognate T cells [20, 35, 66]. Further, immune complexes might activate phagocytes inducing inflammation. Along this line, we found that a mAb against OMGP enhanced phagocyte activation in the presence of OMGP raising the possibility that OMGP-specific Abs might contribute to cortical meningitis.

Together, this study describes OMGP as an autoantigen in a proportion of patients with inflammatory CNS disorders and shows in an animal model that OMGP-specific T cells mediate a novel type of EAE characterized by meningitis above the cortical convexities. Identification of autoimmunity to OMGP might be of future relevance to stratify patients with CNS inflammation.

## Supplementary information

**Additional file 1.** Supplementary Methods. **Fig. S1**. Cell-based assays to detect antibodies to OMGP. **Fig. S2**. Reactivity to OMGP in both CBAs and isotypes of OMGP-specific Abs of patients scored positive. **Fig. S3**. Affinity-purification of OMGP-specific Abs from MS patient 2492. **Fig. S4**. Fluorospot assay identifies MS patients with OMGPspecific T cells. **Fig. S5**. Characterization of new mAbs to OMGP. **Fig. S6**. ELISA to detect sOMGP. **Fig. S7**. Characterization of OMGP-specific T cells. **Fig. S8**. OMGP-specific T cells pave the way for focal demyelination in the cortex. **Fig. S9**. Immune complexes of OMGP and anti-OMGP activate phagocytes. **Table S1**. Subjects used for screening of anti-OMGP Abs. **Table S2**. Clinical characteristics of patients with Abs to OMGP. **Table S3**. Subjects used for analysis of OMGP-specific T cells. **Table S4**. Patients used for quantification of sOMGP in the CSF. References [2, 9, 18, 27, 45, 49, 55–57, 77–80] are cited in additional file 1.

## Data Availability

All data generated or analyzed during this study are included in this article (and its Additional file 1). The datasets generated during and/or analyzed during the current study are available from the corresponding author on reasonable request.
