# Supplementary material for: Oligodendrocyte myelin glycoprotein as a novel target for pathogenic autoimmunity in the CNS
Source: Acta Neuropathol Commun. 2020 Nov 30;8:207. doi: 10.1186/s40478-020-01086-2 (PMC7706210; doi:10.1186/s40478-020-01086-2)
Supplement: Supplementary file 1 — Additional file 1. Supplementary Methods. Fig. S1. Cell-based assays to detect antibodies to OMGP. Fig. S2. Reactivity to OMGP in both CBAs and isotypes of OMGP-specific Abs of patients scored positive. Fig. S3. Affinity-purification of OMGP-specific Abs from MS patient 2492. Fig. S4. Fluorospot assay identifies MS patients with OMGPspecific T cells. Fig. S5. Characterization of new mAbs to OMGP. Fig. S6. ELISA to detect sOMGP. Fig. S7. Characterization of OMGP-specific T cells. Fig. S8. OMGP-specific T cells pave the way for focal demyelination in the cortex. Fig. S9. Immune complexes of OMGP and anti-OMGP activate phagocytes. Table S1. Subjects used for screening of anti-OMGP Abs. Table S2. Clinical characteristics of patients with Abs to OMGP. Table S3. Subjects used for analysis of OMGP-specific T cells. Table S4. Patients used for quantification of sOMGP in the CSF. References [2, 9, 18, 27, 45, 49, 55–57, 77–80] are cited in additional file 1. [file 40478_2020_1086_MOESM1_ESM.docx]

Supplementary Information for

OMGP as a novel target for pathogenic autoimmunity in the CNS

Ramona Gerhards, Lena Kristina Pfeffer, Jessica Lorenz, Laura Starost, Luise Nowack, Franziska S. Thaler, Miriam Schlüter, Heike Rübsamen, Caterina Macrini, Stephan Winklmeier, Simone Mader, Mattias Bronge, Hans Grönlund, Regina Feederle, Hung-En Hsia, Stefan F. Lichtenthaler, Juliane Merl-Pham, Stefanie M. Hauck, Tanja Kuhlmann, Isabel J. Bauer, Eduardo Beltran, Lisa Ann Gerdes, Aleksandra Mezydlo, Amit Bar-Or, Brenda Banwell, Mohsen Khademi, Tomas Olsson, Reinhard Hohlfeld, Hans Lassmann, Tania Kümpfel, Naoto Kawakami, Edgar Meinl

Corresponding author: Prof. Dr. Edgar Meinl

Email: Edgar.Meinl@med.uni-muenchen.de

**This PDF file includes:**

Supplementary Methods

Figures S1 to S9

Tables S1 to S5

SI References

**Methods**

*Cell-based assays to detect antibodies to OMGP*

OMGP-TM, OMGP-GPI (for human, mouse and rat OMGP), and pEGFP-N1 as a control were transiently transfected in HeLa cells. Cells were dislodged 26 h after transfection with ice-cold PBS and 5 x 10^4^ cells per well were analyzed in a 96-well plate. The expression of OMGP was validated with two commercially available Abs to OMGP (R&D systems; MAB1674, AF 1674) (**Fig. S1**); specificity of the latter Ab was confirmed previously by using OMGP deficient mice [11].

For screening, sera were diluted 1:50 (plasma 1:25) in FACS-buffer (PBS and 1 % FCS) and incubated for 45 min on a shaker in the cold room with transfected HeLa cells. For detection of bound antibodies, cells were incubated 1:500 in FACS buffer with goat anti-human-IgG-biotin (Jackson, 109-066-098) for 30 min on the shaker, washed and afterwards incubated 1:2000 in FACS-buffer with Alexa Fluor 647-conjugated streptavidin (Jackson, 016-600-084) for 30 min on the shaker in the dark. In the last step after three times washing, 100 µl of 1:2000 diluted propidium iodide (Sigma, P4864-1ML) was added. Ab binding was analyzed in a flow cytometer (BD FACSVerse) and quantified by using FlowJo software. The gating strategies and the evaluations are explained in detail in **Fig. S1**.

To quantify the binding to OMGP-TM, the gate was set to the highly EGFP-positive cells with fluorescence intensity >500 and we calculated a mean channel fluorescence ratio between the reactivity to OMGP-TM and control vector, basically as we did it to detect MOG-Abs [45,66]. This assay was used for screening. To exclude false positive results, we set a stringent cut-off. This was determined by measurement of 114 healthy control sera and calculated as mean + 6 SDs, resulting in a threshold of 4.4.

The analysis of the binding to OMGP-GPI has to take into consideration that some cells transfected with our ribosome skipping construct, expressed EGFP, but not OMGP (**Fig. 1**). Therefore, quadrants were set with a vertical line at EGFP values >500 to separate the highly transfected cells and the horizontal line was set for each sample individually in the Alexa Fluor 647 channel to include the control cells of the background in pEGFP-N1 transfected cells. This quadrant was then transferred to OMGP-GPI transfected cells and the percentage of both Q2-gates were subtracted (**Fig. S1**).

For the **isotype testing** in sera, assay was carried out as described above, but secondary anti-human-IgG was replaced by 1:50 anti-human-IgG1 (The binding site, AU006) or anti-human-IgG4 (The binding site, AU009) and detected with 1:100 diluted anti-sheep-IgG-Alexa Fluor-647 (Jackson, 713-606-147). Additionally, patient’s purified OMGP antibodies were evaluated for more Ig subclasses. Following antibodies were used 1:50 diluted: anti-human-IgG1-HRP (Zymed, 05-3320), anti-human-IgG2-HRP (Zymed, 05-0520), anti-human-IgG3-HRP (Zymed, 05-3620), anti-human-IgG4-HRP (Zymed, 05-3820), anti-human-IgM-HRP (Zymed, 05-4920). All were detected with 1:100 diluted anti-HRP-Alexa Fluor-647 (Jackson, 123-605-021).

*Generation and characterization of mAbs to OMGP*

Recombinant OMGP, produced via pTT5 vector in HEK-EBNA cells [55], was used for immunization of rats and mice. Hybridoma cells were generated using standard procedures and supernatants were screened for binding to OMGP protein by ELISA. 133 positive supernatants were further validated using OMGP cell-based assays. We stably established three monoclonal antibody clones 22H6 (rat IgG2a/λ), 31A4 (mouse IgG2b/κ) and 14A9 (rat IgG2b/λ) for further analysis. These mAbs show strong reactivity to human OMGP in cell-based assays, biotin-streptavidin ELISA and cross-react to rodent OMGP (**Fig. S5**, **A** and **B**). As secondary antibodies, we used anti-rat-IgG-Alexa Fluor-647 and anti-mouse-IgG-Alexa Fluor-647 (Jackson 212-605-082, 415-605-166). The variable region of 22H6 was cloned and expressed recombinantly with a human IgG1 Fc part, named 22H6-hIgG1. It was selected regarding its specificity and cross reactivity to the rodent protein variants. After replacement of the Fc part from rIgG2A to hIgG1, the antibody retains its specificity and detects OMGP in CBA.

*Detection of circulating antigen-specific B and T cells*

To detect circulating OMGP-specific B cells, PBMCs were differentiated to Ab-secreting plasmablasts using the TLR7/8 agonist resiquimod and IL-2 essentially as described previously [57,71,79]. The presence of OMGP-specific Abs in the supernatant was determined using both OMGP-TM and OMGP-GPI assays.

To detect antigen-specific T cells a recently developed Fluorospot assay was used [8]. Briefly, FluoroSpot plates (FSP-011803, Mabtech) were washed three times with 200 µl sterile PBS and blocked with 200 µl FBS, L-glutamine and pen-strep supplemented RPMI 1640 media (RPMI complete) for 2h at RT. As positive control 0.1 µg/ml of anti-CD3 from the FluoroSpot kit was applied in duplicates to 1.25 x 10^5^ PBMCs per well. 2.5 x 10^5^ PBMCs were seeded in 100 µl and stimulated in duplicates with around 10 beads/cell of OMGP-, Avi-His-tag- and naked CTR beads. Wells were filled with 100 µl RPMI complete and incubated for 44 h at 37 °C with 5 % CO_2_. FluoroSpot assay was developed according the manufacturer’s protocol and spot forming units (SFUs) of plates were analyzed by IRIS reader (Mabtech). At the beginning 53 PBMC donors were included (17 HC, 19 untreated and 17 natalizumab-treated patients), but regarding our three exclusion criteria, 41 remained for further analysis (**Table S3**). First, to guarantee the quality of PBMCs, anti-CD3 response had to induce > 100 IFNγ spot forming units (SFUs), which were not observed in 10 donors. Second, duplicates must be comparable and third no response to naked control beads. These criteria reduced the cohort by two more.

*ELISAs*

To detect antibodies against human OMGP, recombinantly produced OMGP was enzymatically biotinylated on its Avi-tag and used for coating streptavidin plates (Thermo Scientific, 436014). Sera were diluted 1:100 in PBST (0.05 %), added to OMGP-coated plates and incubated on a shaker at 4 °C overnight. Streptavidin wells without OMGP were used as controls. On the next day 1:7000 diluted secondary goat anti-human-IgG-HRP (Jackson, 109-036-003) was added, the plate was developed with A&B-ELISA detection solution (R&D, DY999) and stopped with 1 M H_2_SO_4_. The OD was measured at 450 nm and 540 nm. To detect the anti-MOG mAb 8-18C5-hIgG1 with a human IgG1 Fc in the blood of EAE rats, biotinylated MOG was used for coating, rat sera were diluted 1:20 and detected by same anti-human-IgG-HRP. To quantify C1q-binding of OMGP-specific Abs, biotinylated OMGP was bound to streptavidin plates (Thermo scientific, 436014). Subsequently Abs, C1q complement (Sigma, C1740-5MG), and an anti-C1q-HRP antibody (LSBio, C41845) were added. Assays were developed with TMB (Sigma, T8665-100ML) as a substrate.

*Proteomic sample preparation and LC-MSMS measurement*

Each 20µl of CSF were digested using a modified FASP procedure [80]. LC-MS/MS analysis was performed on a Q-Exactive HF mass spectrometer (Thermo Scientific) online coupled to an Ultimate 3000 nano-RSLC (Thermo Scientific). Tryptic peptides were automatically loaded on a C18 trap column (300 µm inner diameter (ID) × 5 mm, Acclaim PepMap100 C18, 5 µm, 100 Å, LC Packings) at 30 µl/min flow rate prior to C18 reversed phase chromatography on the analytical column (nanoEase MZ HSS T3 Column, 100 Å, 1.8 µm, 75 µm x 250 mm, Waters) at 250 nl/min flow rate in a 95 minutes non-linear acetonitrile gradient from 3 to 40 % in 0.1 % formic acid. Profile precursor spectra from 300 to 1500 m/z were recorded at 60000 resolution with an automatic gain control (AGC) target of 3e6 and a maximum injection time of 50 ms. TOP10 fragment spectra of charges 2 to 7 were recorded at 15000 resolution with an AGC target of 1e5, a maximum injection time of 50 ms, an isolation window of 1.6 m/z, a normalized collision energy of 27 and a dynamic exclusion of 30 seconds.

Generated raw files of two individual datasets of CSF (altogether 20 CSF samples) were analyzed using each Progenesis QI for proteomics (version 3.0, Nonlinear Dynamics, part of Waters) as described previously [27]. Features of charges 2-7 were used and all MSMS spectra were exported as mgf file. Peptide searches were performed using Mascot search engine (version 2.6.1) against the Swissprot Human protein database (20194 sequences, 11329970 residues). Search settings were: 10 ppm precursor tolerance, 0.02 Da fragment tolerance, one missed cleavage allowed. Carbamidomethyl on cysteine was set as fixed modification, deamidation of glutamine and asparagine allowed as variable modification, as well as oxidation of methionine. Applying the percolator algorithm [9] resulted in a peptide false discovery rate (FDR) of 0.49 % or 0.40 %. Search results were reimported in the Progenesis QI software.

*Culture and staining of primary cell cultures and spinal cord tissue*

Hippocampi and cortices from embryonic (E16) mice were collected and incubated in digestion medium (DMEM containing 200 U papain and 1 mg/ml L-cysteine, pH 7.4) at 37 °C for 20 minutes. Digested tissues were then mechanically dissociated in plating medium (DMEM containing 1 0% FBS and 1 % pen/strep) and plated on glass coverslips coated with 25 µg/ml of poly-D-lysine at a density of 100,000 cells per well/12-well plate. 2-4 hours after plating, medium was exchanged into Neurobasal medium (Gibco) supplemented with B27 (Gibco), 1 % pen/strep, and 5 mM GlutaMAX (Gibco). Neurons were maintained at 37°C in a cell culture incubator with 5 % CO2 before fixation with ice-cold absolute methanol at day-in-vitro 7 (DIV7). For staining, fixed coverslips were blocked for 1 h at RT in 5 % sucrose and 10 % FCS in PBS. Subsequently, primary antibodies diluted in blocking buffer were added at 4 °C overnight: anti-β-III-tubulin 1:200 (Cell signaling, D7169) and anti-OMGP 22H6-rIgG2a with 20 µg/ml. On the next day, cover slips were washed three times with 200 µl PBS. Secondary antibodies anti-rat-IgG-Alexa Fluor-488 (Invitrogen, A-21208) and anti-rabbit-IgG-Alexa Fluor-594 (Invitrogen, A-21207) were diluted 1:500 in blocking buffer incubated for 1 h at RT. Nuclei were stained with DAPI (Thermo Scientific, 62248), mounted with Vectashield (Biozol, VEC-H-1000) and slides sealed. Neurons were imaged using the Leica DFC 300 G microscope.

Murine oligodendrocyte precursor cells (OPCs) were isolated by immunopanning from spinal cord and cerebrum of 6-9 days old mice. The differentiation was induced by 10 ng/ml of growth factor CNTF (Peprotech, 450-13) [18,77,2]. Cells were stained 24 h after induction with an Ab to O4 marker and after 48 h with an Ab to MBP. For O4 staining, cells were fixed with 4 % PFA at RT; for MBP staining, cells were permeabilized with 0.5 % Triton X-100 after fixation. Then cover slips were blocked with 300 µl 5 % FCS in PBS for 1 h at RT and incubated with primary antibodies in blocking buffer at 4 °C overnight: 10 µg/ml of rabbit anti-MBP (abcam, ab40390), 10 µg/ml of mouse anti-O4 (R&D, MAB1326) and 20 µg/ml of 22H6 anti-OMGP. On the next day, cells were washed as mentioned and incubated for 1 h at RT with 10 µg/ml of secondary antibodies: anti-mouse-IgG-Alexa Fluor-488 (Invitrogen, A-21202), anti-rabbit-IgG-Alexa Fluor-488 (Invitrogen, A-11008) and anti-rat-IgG-Alexa Fluor-594 (Invitrogen, A-11007). Cover slips were washed, mounted and fixed with Roti Mount FluorCare DAPI (Roth, HP20.1). Human induced pluripotent stem cell (iPSC) were used to generate O4^+^ oligodendrocytes (hiOL) as described in previous studies [17,78]. At day 21 of differentiation, hiOL were detached by treatment with accutase and subsequently fixed in 0.4 % PFA. After one washing step, anti-OMGP mAb 22H6 (10 µg/ml) and anti-O4-APC (Miltenyi) (1:50) were simultaneously applied for 30 min at 4°C. After washing, secondary antibody anti-human-IgG-biotin was added for another 30 min at 4°C, followed by an additional washing step and application of streptavidin FITC for 30 min at 4°C allowing for detection. Afterwards, cells were analyzed in FACSAria II cell sorter (BD Biosciences). OMGP^+^/O4^+^ cells were identified by utilizing unstained cells as well as isotype and secondary antibody controls.

PFA fixed (4 %) and cryoprotected (30 % sucrose) spinal cord of C57BL/6J mice was sliced sagittal with 55 µm at the cryostat. Free-floating stainings were carried out in 24-well plates, washed in PBS at RT, treated with 10 mM sodium citrate pH 8.5 for 20 min at 80 °C and transferred into blocking buffer (10 % goat serum in 0.5 % Triton-PBS). Primary Abs were incubated over night in 1 % goat serum in 0.5 % Triton/PBS: anti-β-III-tubulin 1:200 (Cell signaling, D7169) and anti-OMGP 22H6-rIgG2a with 20 µg/ml. After washing, tissue was incubated with secondary Abs diluted 1:1000 in the same buffer: anti-rabbit-IgG-Alexa Fluor-488 (Invitrogen, A-11008) and anti-rat-IgG-Alexa Fluor-594 (Invitrogen, A-11007). On the next day, samples were washed, nuclei were stained with DAPI (Thermo Scientific, 62248), mounted with Vectashield (Biozol, VEC-H-1000) and slides sealed. Tissue was scanned on an upright FV1000 confocal microscopy system (Olympus) with × 10/0.4 air, × 20/0.85 and × 60/1.42 oil immersion objectives.

*EAE transfer – flow cytometric characterization of T cells*

T cells were stimulated with their specific antigen which was presented by 50 Gy irradiated thymocytes. Two days later, the T cells were purified by Nycoprep gradient as following. T cells were suspended DMEM supplemented with 25mM HEPES (pH=7.4) and overlaid on Nycoprep (14.1% Nycodenz, 0.44% NaCl, 5mM Tricine/NaOH (pH=7.2)). After centrifugation at 800g for 10 minutes, activated T cells were collected from interface and used for further staining.

For cell surface staining, T cells were stimulated with their specific antigen for 2 days and expanded in DMEM supplemented with 10 % Horse serum and 2 % Concanavalin A stimulated EL4IL2 cell supernatant. Following antibodies were used 1:100 dilution in FACS buffer (PBS with 1% Rat serum and 0.05 % NaN3, Isotype control (MOPC31, Sigma), CD4 (W3/25, AbD Serotec), CD8a (OX-8, Biolegend), αβTCR (R73, AbD Serotec), CD25 (OX-39, AbD Serotec), CD134 (OX-40, AbD Serotec), CD45RA (OX-33, Biolegend), CD45RC (OX-22, AbD Serotec), CD44 (OX-50, AbD Serotec), CD11a (wt.1, purified from hybridoma), CD11b (OX-42, AbD Serotec), CD49d (TA-2, Invitrogen). The binding of antibodies was detected by goat anti-mouse IgG-APC (1:400, Jackson laboratories).

For intracellular staining, T cells were stimulated with their specific antigen for 2 days and purified by Nycoprep gradient as following. T cells were suspended DMEM supplemented with 25mM HEPES (pH=7.4) and overlaid on Nycoprep (14.1% Nycodenz, 0.44% NaCl, 5mM Tricine/NaOH (pH=7.2)). After centrifugation at 800g for 10 minutes, activated T cells were collected from interface. The T cells were incubated with 5 µg/ml Brefeldine alone for 2 hours or 100ng/ml PMA/100nM ionomycin for 2 hours followed by 5 µg/ml Brefeldine for 2 hours. After incubation, the T cells were fixed with 2% PFA by incubating for 15 min on ice and stored in PBS further staining. The T cells were permeabilized by incubating with permeabilization buffer [1] for 20 min on ice. Following antibodies were used in permeabilization buffer: mouse anti-rat IFNγ (1:100, DB1, eBioscience), mouse isotype control (MOPC31, 1:100, Sigma), rat anti-mouse/rat IL17-PE (1:400, TH11-18H10, BD) and rat isotype control–PE (1:400, R-3-34, BD). The binding of anti-IFNγ antibody was detected by goat anti-mouse IgG-APC (1:400, Jackson laboratories).

*Histopathology of EAE rats and quantification*

The histopathology was done basically as described in [56,49,66]. Primary Abs against the following targets were used: CD3 (T cells; rabbit monoclonal; Neomarkers, RM-9107-5; 1:2,000), ED1 (CD68, phagocytic macrophages and microglia; mouse monoclonal; Serotec, MCA341R, 1:10,000), Iba 1 (panmicroglia and macrophages; rabbit polyclonal; Wako, 019-19741; 1:3,000), cyclic nucleotide phosphodiesterase (oligodendrocytes; mouse monoclonal; Sternberger Monoclonals, BioLegend, SMI 91; 1:2,000), glial fibrillary acidic protein (astrocytes; rabbit polyclonal; Dako, Z0334; 1:3,000), AQP4 (Rabbit polyclonal antibody, Sigma-Aldrich), MOG (8-18C5), rat-Ig (biotinylated; GE Healthcare/Amersham) human Ig (biotinylated species specific anti-human Ig; donkey polyclonal, Jackson ImmunoResearch, 709-065-149; 1:1,000) and activated complement (C9neo antigen, rabbit polyclonal; 1:2,000).

Lymphocyte infiltration in the meninges was quantified in sections stained with hematoxylin & eosin in the microscope at an objective magnification of 20 x. The length of the microscopic field was spanned by cortical meninges over a distance of 0.615 mm. The number of lymphocytes in the meninges was determined in 6 microscopic fields per animal and finally calculated as lymphocytes / mm of meningeal length. To validate that the counts of meningeal lymphocytes corresponded to T-cells we performed the same counting procedure in sections of 9 animals stained by immunocytochemistry for CD3. The counts between sections stained for H&E and CD3 varied by less than 5 % per animal. The values in the figure are presented as lymphocytes per mm length of cortical meninges.

Spinal cord inflammation was assessed by counting the number of perivascular inflammatory infiltrates in on average 20 spinal cord cross sections covering all cord segments per animals. In a second step the average area of spinal cord cross sections was determined in the same sections. The values in the figure represent the average number perivascular inflammatory infiltrates per mm^2^ of spinal cord tissue.

*Phagocyte activation*

To analyze whether anti-OMGP induces activation of phagocytes in the presence of its antigen, HeLa cells were transiently transfected with OMGP-TM and 2 x 10^4^ of these transfected cells were seeded in a 96-well flat-bottom along with 5 x 10^5^ THP-1 cells. Then either the anti-OMGP mAb 22H6-hIgG1 or as control the MOG-specific mAb with the same isotype, 8-18C5-hIgG1, were added. After 17 h supernatant was collected and IL-8 production was measured by ELISA (R&D, DY208-05).


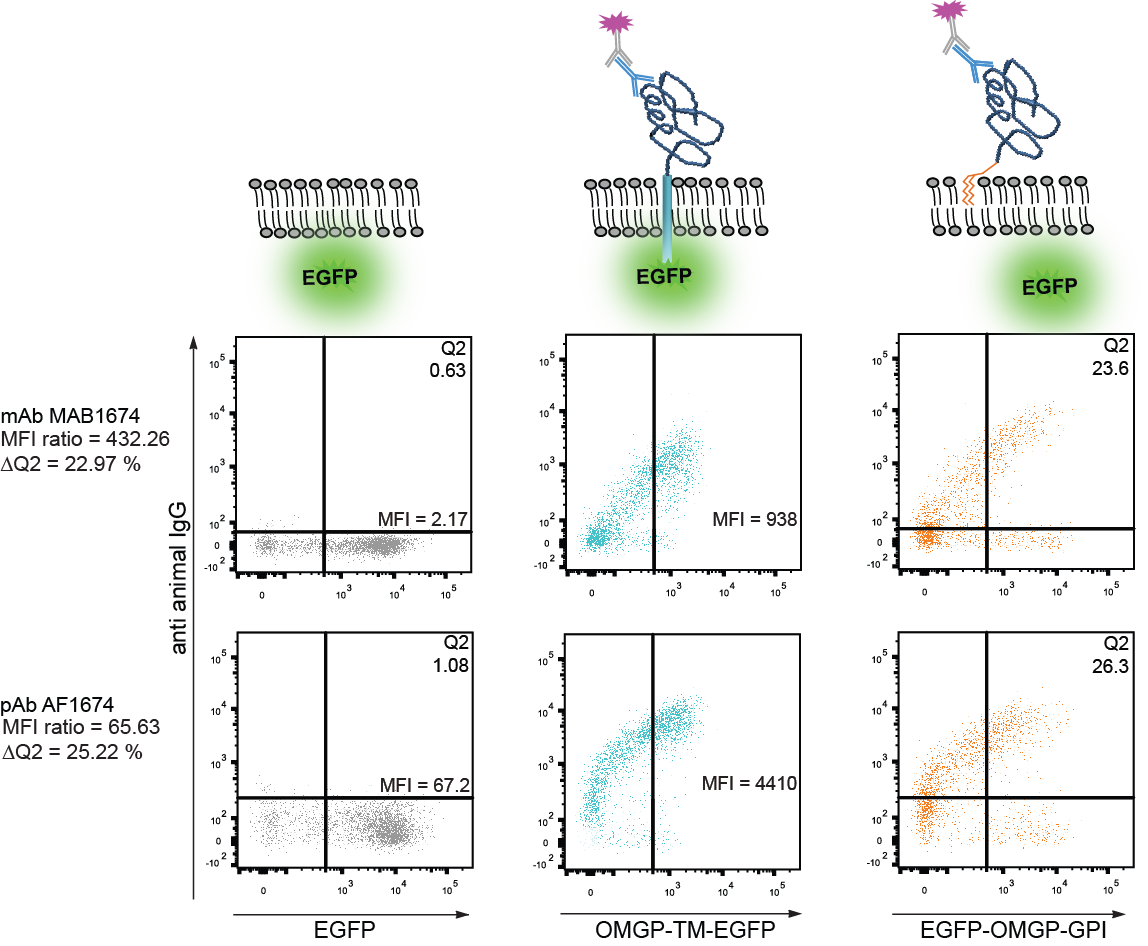


## Fig. S1. Cell-based assays to detect antibodies to OMGP.

Two cell-based assays (CBA) were developed to detect Abs against OMGP. The upper row shows the cartoons of proteins expressed in HeLa cells after transient transfections: EGFP alone (left), OMGP displayed as a fusion protein with the transmembrane (TM) part of CD80 and EGFP (middle), OMGP anchored in the membrane with its natural GPI anchor and EGFP as a separate protein due to a ribosome skipping element in our construct (details in materials and methods). The middle and bottom rows show the results with two commercially available anti-OMGP Abs, MAB1674 (middle) and AF1674 (bottom). The OMGP-TM CBA results are shown with blue, the OMGP-GPI CBA in orange and the EGFP expressing cells in grey. The calculation of the binding to OMGP was done as follows. OMGP-TM-EGFP: We gated on the EGFP-high expressing cells (>500 EGFP, right from the vertical line in the left and middle column) and calculated the mean fluorescence intensity (MFI) ratio of the reactivity to OMGP-TM-EGFP and EGFP alone. EGFP-OMGP-GPI: In addition to the vertical line (gating on EGFP high expressing cells), a horizontal line is introduced, adapted to the EGFP background signal. We then calculated ΔQ2 by subtracting the Q2 value of the EGFP control from the Q2 value of EGFP-OMGP-GPI. For illustration, the measured numbers and the calculations are included in this figure.


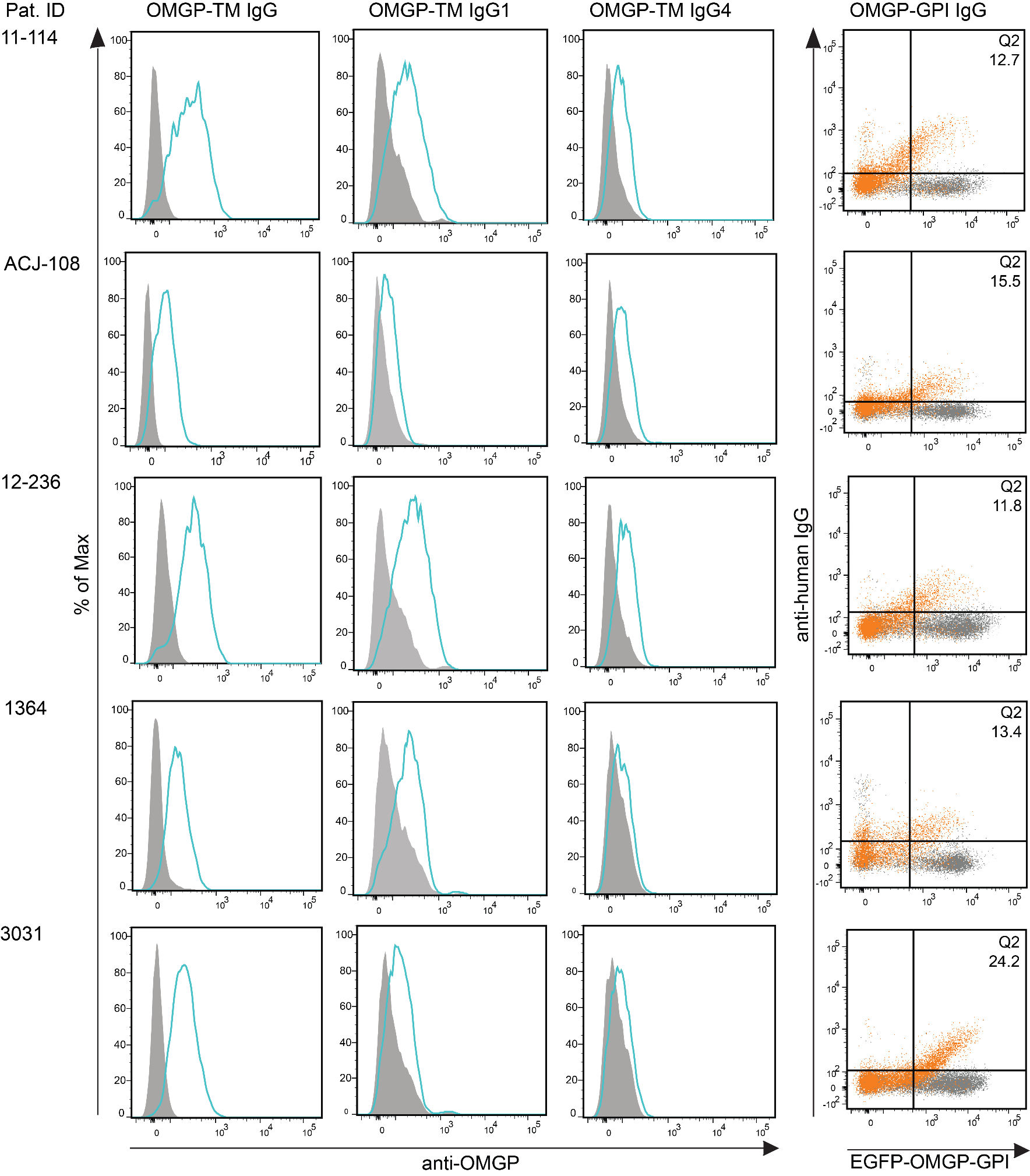


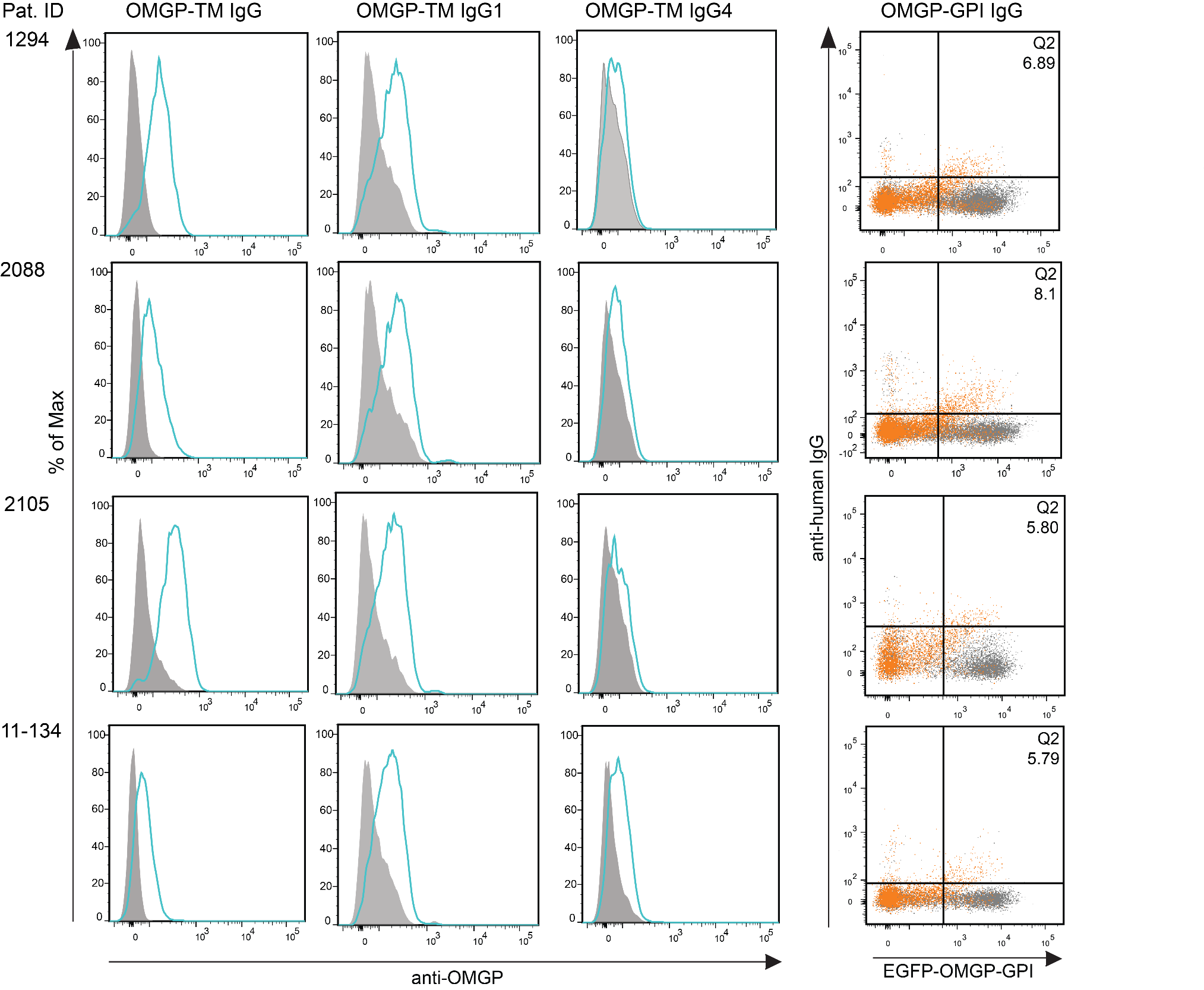


## Fig. S2. Reactivity to OMGP in both CBAs and isotypes of OMGP-specific Abs of patients scored positive.

The patient with the highest reactivity is presented in detail in **Fig. 2**, OMGP-reactivity of the other nine patients who scored positive are shown here. The left three columns show the reactivity to OMGP in the cell-based assay with OMGP-TM-EGFP; closed gray graphs represent the recognition of cells transfected with EGFP; the blue open lines show the reactivity to cells transfected with OMGP-TM-EGFP. The column on the right shows the reactivity to GPI-anchored OMGP; the gray dots show the reactivity to cells transfected with EGFP; the orange dots show the reactivity to cells transfected with EGFP-OMGP-GPI. The numbers in the upper right quadrant give the Q2 value as explained in **Fig. S1**.


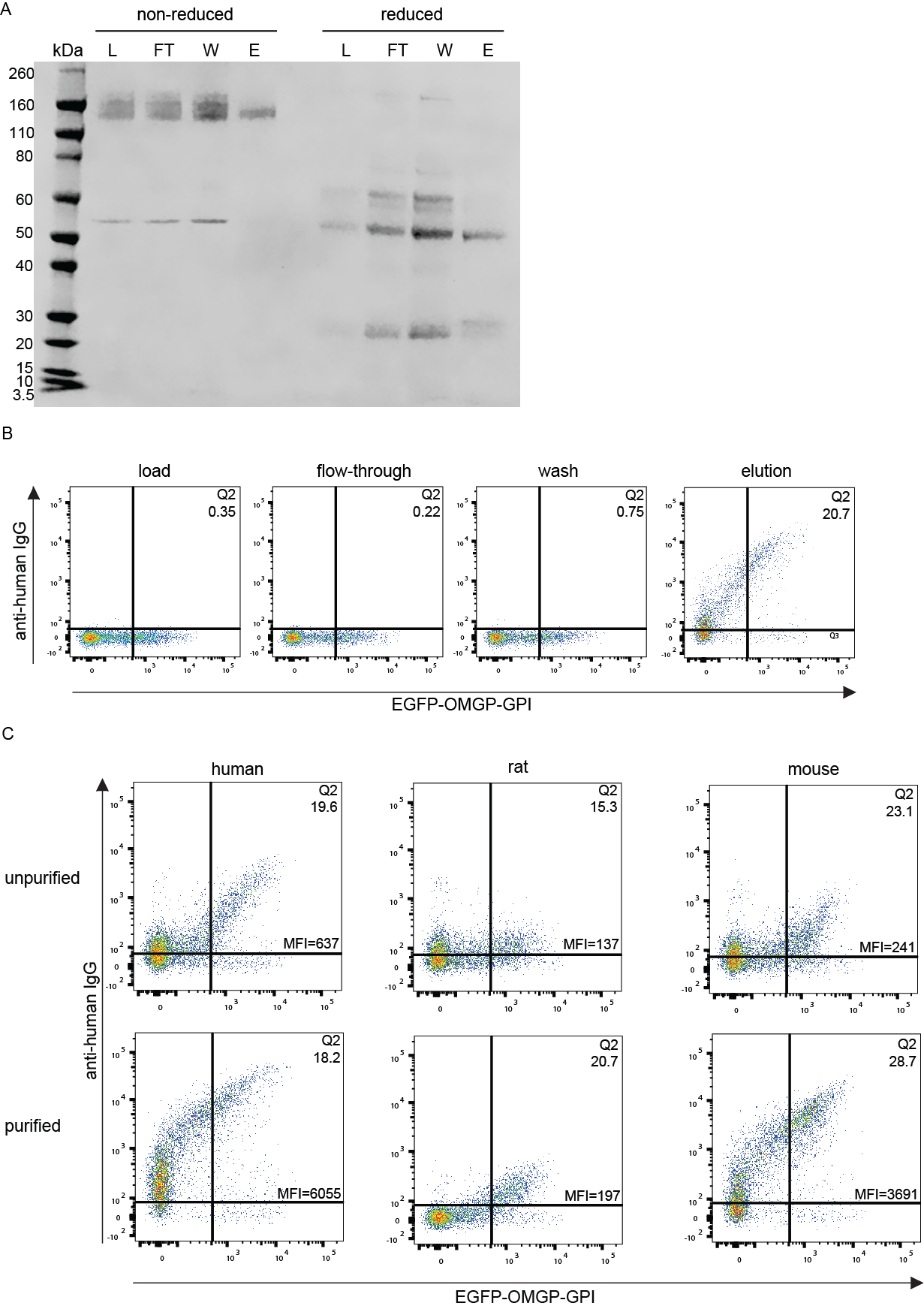


## Fig. S3. Affinity-purification of OMGP-specific Abs from MS patient 2492.

(**A**) Plasma from patient 2492 underwent ammoniumsulfate precipitation and subsequent affinity-purification on a streptavidin column loaded with biotinylated OMGP. The different stages of this affinity-purification load (L), flow-through (FT), wash (W) and elution (E) were separated by SDS-PAGE under reducing and non-reducing conditions and subsequently stained by Coomassie. In the E fraction the pure IgG is visible; under non-reducing conditions only the IgG with 150 kDa is visible, while heavy chain above 50 kDa and light chain at 25 kDa are visible under reducing conditions. (**B**) The four fractions were analyzed for binding to OMGP using our OMGP-GPI CBA. In each of the four fractions, the same concentration of IgG (50 ng IgG per 5 x 10^4^ EGFP-OMGP-GPI transfected HeLa cells) was applied. (**C**) Cross-reactivity to rat and mouse OMGP. The unpurified material (plasma diluted 1:50) and the purified Abs (50 ng of IgG) were added to 5 x 10^4^ HeLa cells. In addition as a quantitative value, the mean fluorescence intensity (MFI) is shown below the percentage of cells in Q2 to indicate the intensity of binding.


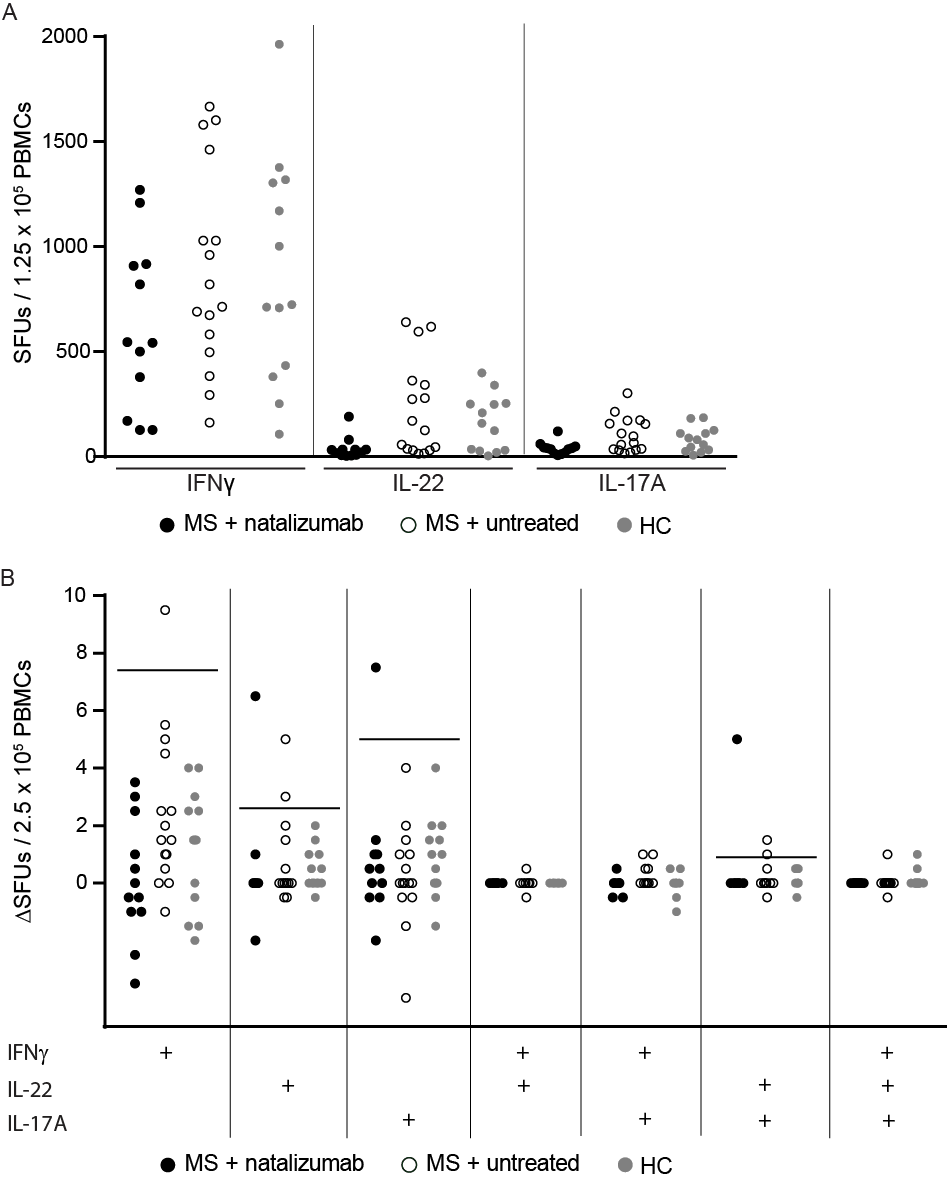


## Fig. S4. Fluorospot assay identifies MS patients with OMGP-specific T cells.

Filled black circles represent MS + natalizumab (n=12) treated patients, open black circles display MS + untreated patients (n=16) and grey symbols HCs (n=13). Each symbol represents the mean of two technical replicates. (**A**) IFNγ, IL-22 and IL-17A response after anti-CD3 stimulation. The spot forming units (SFUs) of CD3 are calculated from 1.25 x 10^5^ PBMCs in the stimulation well. (**B**) The response to OMGP is given as ΔSFUs per 2.5 x 10^5^ PBMCs, which was calculated as number of spots in response to OMGP-coated beads minus number of spots in response to Avi-His-peptide coated beads. At the x-axis the cytokines analyzed by single -, double -, or triple-staining are indicated. Means of the HC values plus 3 SDs were calculated and indicated as a horizontal line, if at least one sample was above: 7.4 for IFNγ, 2.6 for IL-22, 5 for IL-17A and 0.9 for IL-22^+^/IL-17A^+^ assay. The three symbols in the natalizumab treated group, scoring positive above the threshold are coming from the same donor. The five values scoring positive in the untreated group are coming from four different patients.


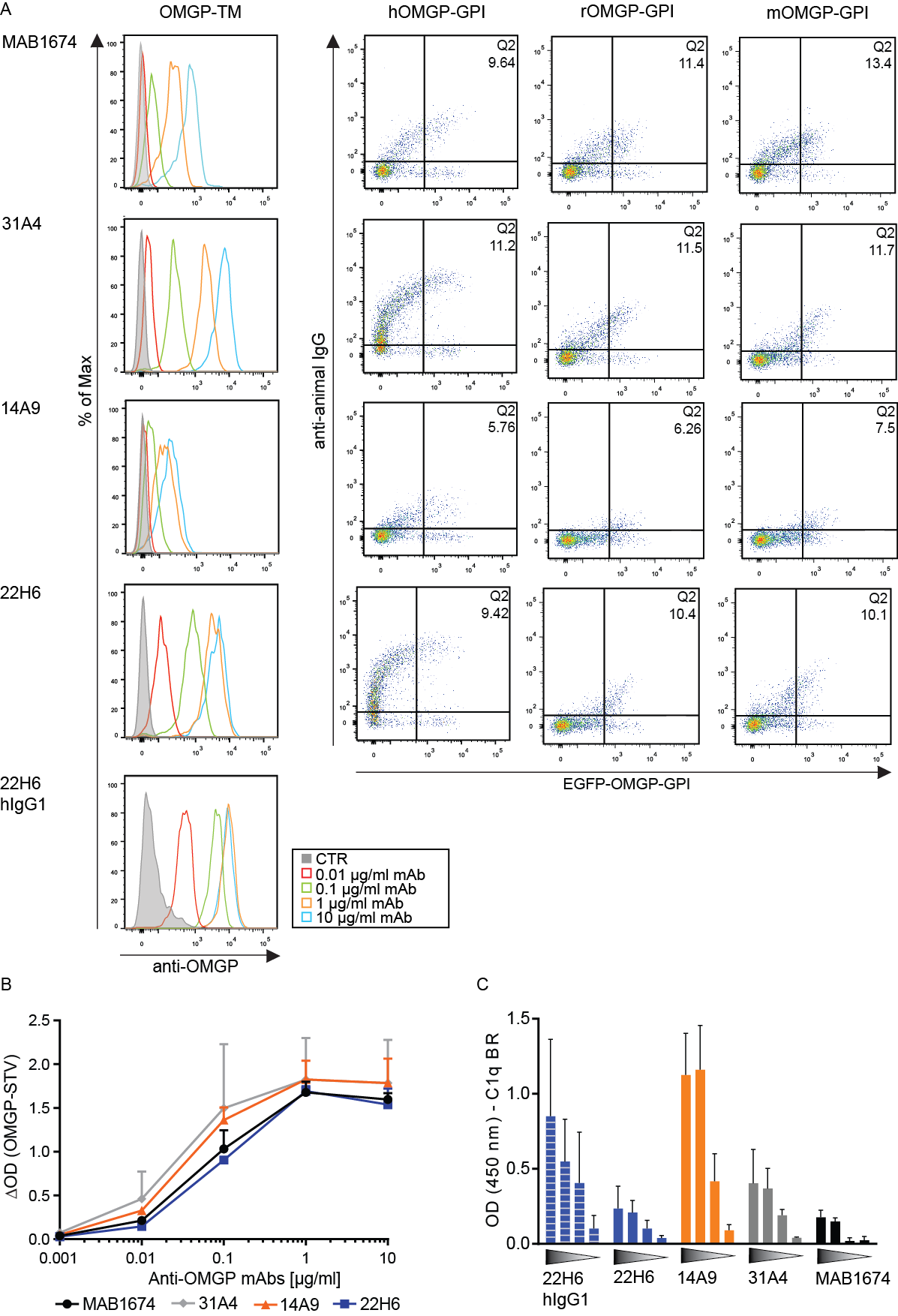


## Fig. S5. Characterization of new mAbs to OMGP.

We have generated three new mAbs to OMGP, 22H6 (rat IgG2a), 14A9 (rat IgG2b) and 31A4 (mouse IgG2b). Further, we have cloned 22H6 to produce it recombinantly with a human IgG1 Fc, 22H6-hIgG1. These mAbs are compared with the commercially available MAB1674 (rat IgG1). (**A**) Serial dilution of these mAbs were tested in our OMGP-TM assay (left). The closed gray graph (CTR) represents the binding of 10 µg/ml of corresponding mAb to cells transfected with EGFP, the open colored lines show the binding of the indicated concentrations of the mAbs to cells transfected with OMGP-TM-EGFP. The mAbs were also tested with 1 µg/ml for recognition of human, mouse, and rat OMGP using our EGFP-OMGP-GPI assay. The values in the upper right quadrant indicate the ΔQ2 (explained in **Fig. S1**). (**B**) Recognition of OMGP by ELISA. Streptavidin plates were coated with biotinylated human OMGP and the mAbs were added at the indicated concentrations. To obtain the ΔOD, reactivity to the streptavidin coated wells was subtracted. Values indicate mean of two replicates and error bars show SEM. (**C**) Analysis of C1q-binding of these mAbs as described in materials and methods. C1q background signal is subtracted from each measurement. Triangle represents decreasing mAb concentrations 10/1/0.1/0.01 µg/ml. Bars display mean of two replicates with SEM.


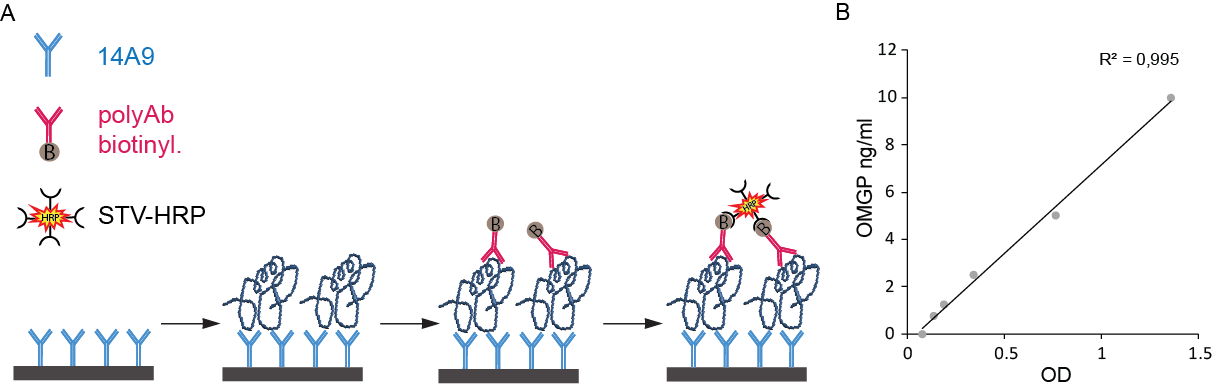


## Fig. S6. ELISA to detect sOMGP.

(**A**) Scheme of the ELISA. Our new mAb 14A9 was used for coating and the bound sOMGP was detected with the commercially available polyclonal Ab AF1674, which we had biotinylated. (**B**) Standard curve of this ELISA. OD was calculated by subtraction of 540 nm plate background values from values at 450 nm.


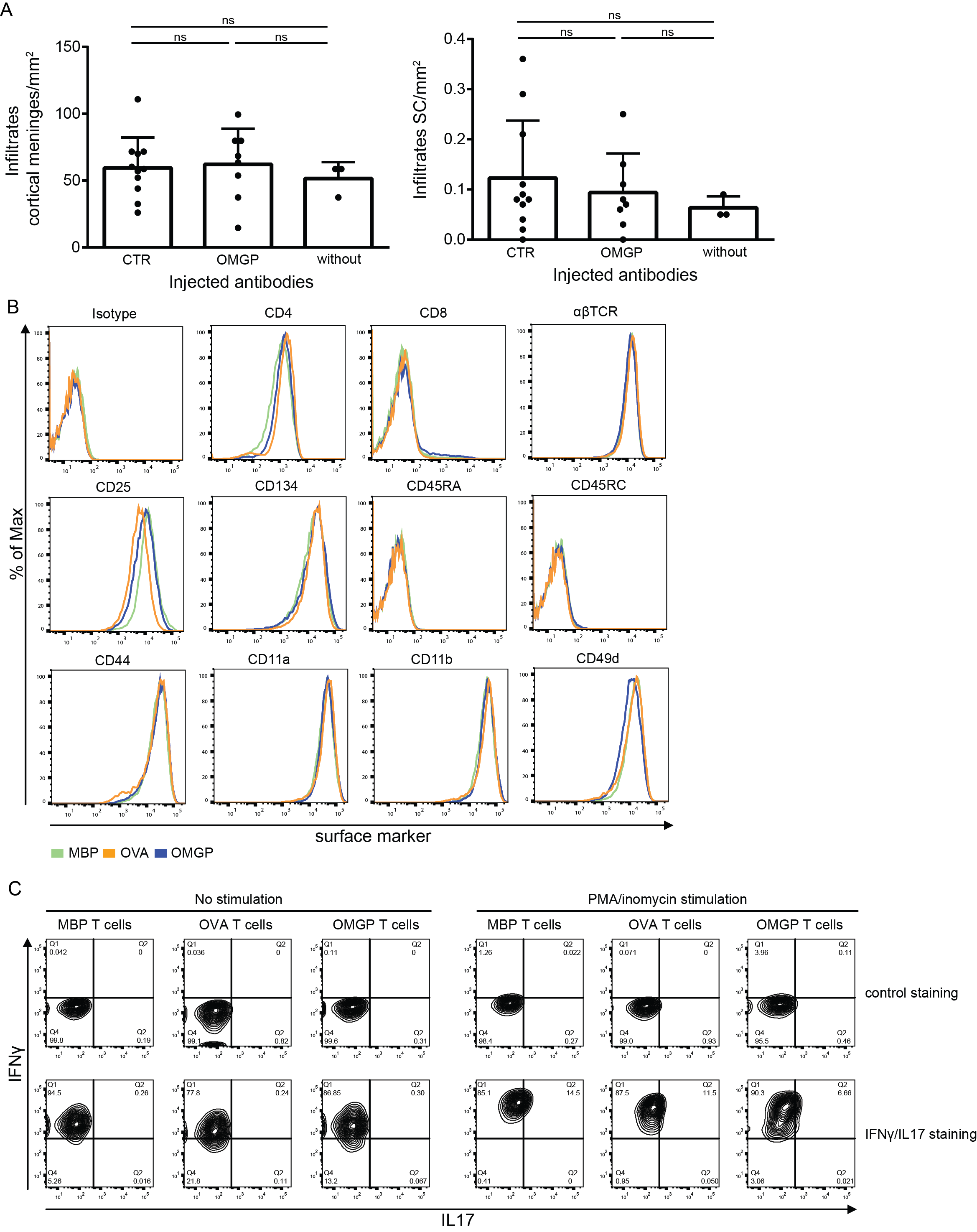


## Fig. S7. Characterization of OMGP-specific T cells.

(**A**) Quantification of the infiltrates in meninges of the cortex and in the spinal cord (SC) after transfer of OMGP-specific T cells in the presence or absence of Abs. OMBP-specific T cells were injected into Lewis rats, two days later the indicated Abs and after another three days, the animals were sacrificed for histological analysis. The infiltration in these three groups did not show a significant difference (Tukey's honest significance test). (**B**) T cells were stimulated with their respective antigen for 2 days and analyzed by flow cytometry for the indicated surface markers. OMGP-specific T cells (blue) are compared to MBP- (green) and OVA-specific (orange) T cells. All T cells expressed CD4, αβTCR, activation markers (CD25, CD134) and adhesion proteins (CD44, CD11a/b, CD49d), whereas they were negative for naïve T cell markers (CD45RA, CD45RC). (**C**) The intracellular flow cytometry staining of IFNγ (y-axis) and IL17 (x-axis) was conducted on fixed and permeabilized T cells after 2 days of stimulation with their antigen. In the right panel, cells were additionally stimulated with PMA/ionomycin for 2h prior staining. Control staining represents IFNγ/IL17 isotype control stained cells to establish the gating.


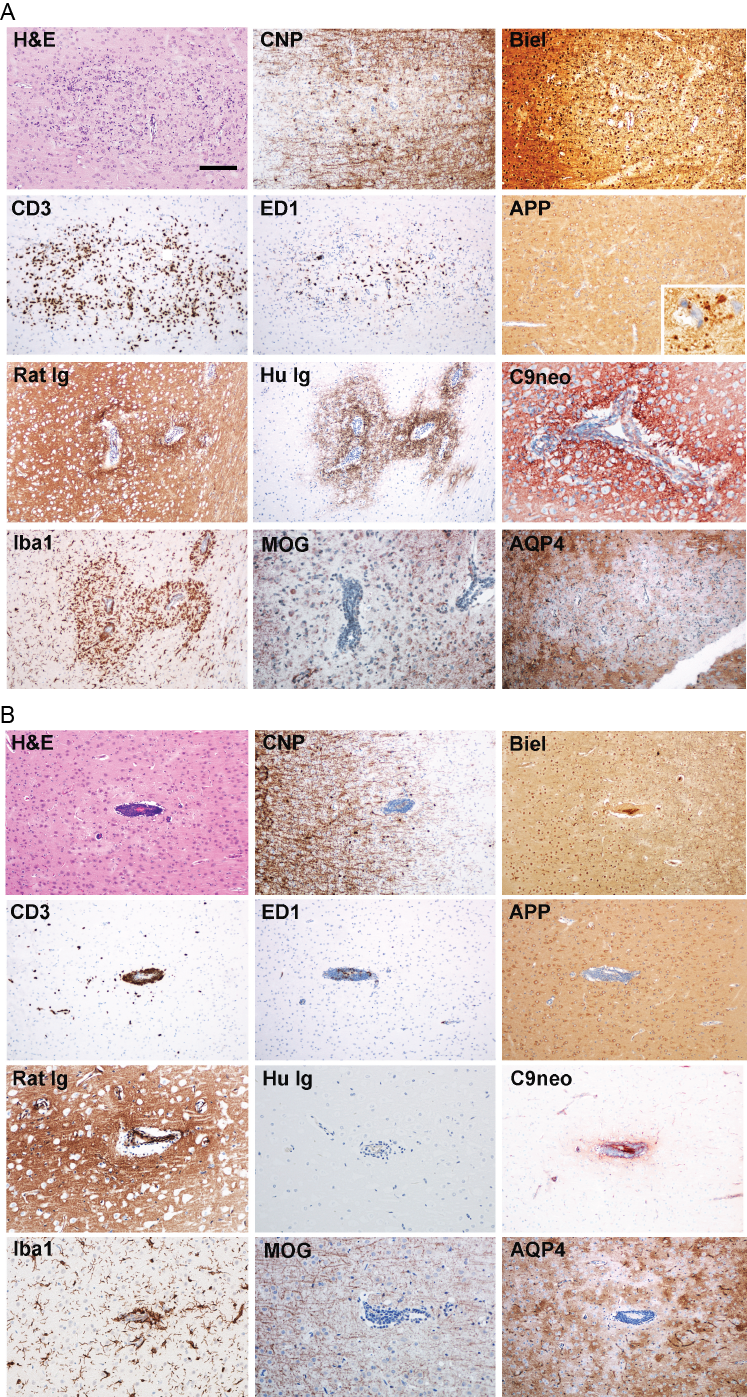


## Fig. S8. OMGP-specific T cells pave the way for focal demyelination in the cortex.

Lewis rats were injected with OMGP-specific T cells, two days later either the MOG-Ab 8-18C5-hIgG1 (**A**) or human control Ig (**B**) was given and after three more days, the animals were sacrificed. Demyelination in the outer cortical layers is only seen, when sensitive immunocytochemical stains are used, since staining with LFB is not sufficiently sensitive to visualize the very thin myelin sheaths in the outer cortical layers. Sections of the cortex were stained with H&E, Bielschowsky's Silver Staining (Biel) or immunohistochemically for the indicated targets. Scale bar represents 100 µm.


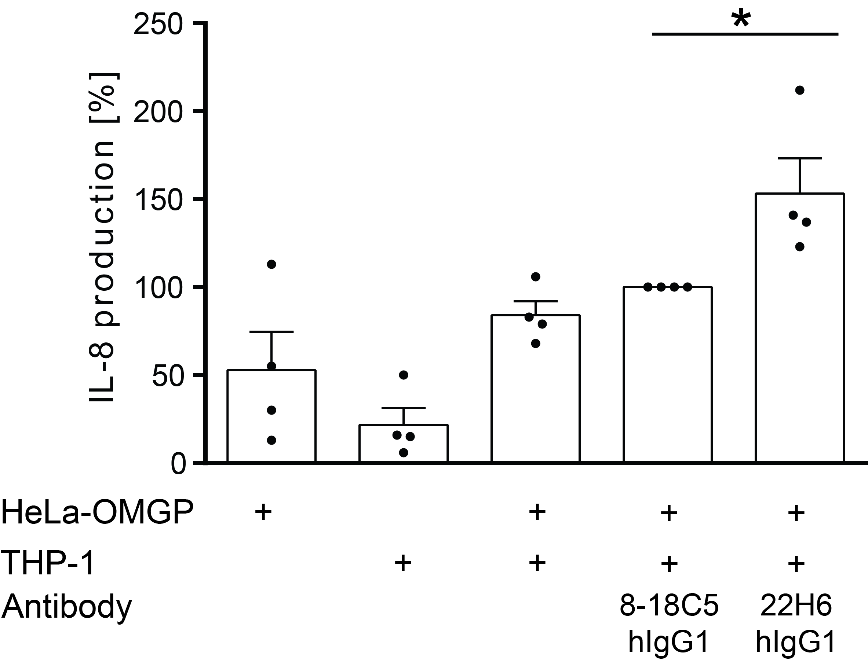


## Fig. S9. Immune complexes of OMGP and anti-OMGP activate phagocytes.

OMGP expressing HeLa cells were incubated with the THP-1 monocytic cell line with the anti-OMGP mAb 22H6-hIgG1 or the anti-MOG mAb 818C5-hIgG1 as indicated. IL-8, released in the supernatant in the next 17 hours, was analyzed by ELISA. Four independent experiments were performed, the secreted IL-8 together with the control mAb to MOG (8-18C5-hIgG1) was set as 100 % in each individual experiment and the IL-8 secretion in the other conditions was calculated. Each symbol represents the mean of two technical replicates of an individual experiment and error bars indicate SEM. (* p ≤ 0.05, unpaired t-test).

## Table S1. Subjects used for screening of anti-OMGP Abs.

| Cohort | Number | Mean age | Male | Female |
| --- | --- | --- | --- | --- |
| MS/CIS | 352 | 38.9 (+/- 11.6) | 125 | 227 |
| ADEM | 28 | 8.1 (+/-4.4) | 15 | 13 |
| NINDC | 45 | 37.9 (+/- 13.7) | 23 | 22 |
| INDC | 30 | 48.1 (+/- 13.2) | 7 | 23 |
| NMOSD | 10 | 40.8 (+/- 11.5) | 5 | 5 |
| MOGAD | 9 | 42.8 (+/- 10.6) | 6 | 3 |
| HC | 114 | 33.6 (+/- 11.5) | 42 | 72 |
|  | 588 |  |  |  |

Multiple sclerosis (MS), clinical isolated syndrome (CIS), acute disseminated encephalomyelitis (ADEM), non inflammatory neurological disease control (NINDC), inflammatory neurological disease control (INDC), neuromyelitis optica spectrum disorder (NMOSD), MOG antibody-associated disease (MOGAD), healthy controls (HC).

## Table S2. Clinical characteristics of patients with Abs to OMGP.

| **#** | **Sex** | **Age** | **Diagnosis** | **Clinical description** | **EDSS** |
| --- | --- | --- | --- | --- | --- |
| **Munich MS** | | | | | |
| 2492 | M | 37 | RRMS | Relapses: 4 (brainstem, myelitis, ON)  Lesions: brainstem, SC multiple short, cerebral MS typical (deep WM and juxtacortical) 🡪 brain atrophy  Disease duration 17.7 years, OMGP seropositive in 60 month follow up | 2.5 |
| 1294 | F | 48 | RRMS | Relapses >10 (brainstem, recurrent myelitis, ON, cognitive dysfunction)  Lesions: brainstem, SC multiple short, cerebral multiple MS lesions (deep WM and cortical) 🡪 brain atrophy  Disease duration 26.6 years, OMGP seropositive in 48 month follow up | 6.0 |
| 2088 | M | 51 | RRMS | Relapse: 1 (myelitis, pathological VEP)  Lesions: deep WM, LETM 🡪 brain atrophy  Disease duration 0.8 years | 2.5 |
| 2105 | M | 58 | RRMS | Relapse: 1 (myelitis)  Lesions: SC multiple, several WM and subcortical  Disease duration 0.6 years | 2.5 |
| 1364 | F | 37 | CIS/MS | Relapse: 1 (large tumefactive cerebral lesion)  Lesions: large parietal contrast enhancing deep WM  Disease duration 2.5 years | 1.5 |
| 3031 | F | 36 | CIS/MS | Relapse: 1 (myelitis)  Lesions: SC short, deep WM  Disease duration 0.5 years | 0.0 |
| **Stockholm** | | | | | |
| 11-114 | M | 45 | SPMS | more than nine lesions in MRI scan | 4 |
| 11-134 | F | 29 | RRMS | remission, more than nine lesions in MRI scan | 2 |
| 12-236 | M | 29 | Psychosis | no oligoclonal bands detected, no lesions in MRI | / |
| **Canadian pediatric** | | | | | |
| ACJ-108 | F | 10.5 | ADEM | monophasic acquired demyelinating syndrome | / |

Multiple sclerosis (MS), relapsing remitting multiple sclerosis (RRMS), clinical isolated syndrome (CIS), optic neuritis (ON), acute disseminated encephalomyelitis (ADEM), white matter (WM), spinal cord (SC), visual evoked potentials (VEP), longitudinally extensive transverse myelitis (LETM), expanded disability status scale (EDSS).

## Table S3. Subjects used for analysis of OMGP-specific T cells.

| Cohort | Number | Mean age | Male | Female |
| --- | --- | --- | --- | --- |
| MS + natalizumab | 12 | 38.8 (+/- 11.2) | 3 | 9 |
| MS + untreated | 16 | 44.0 (+/- 13.6) | 5 | 11 |
| HC | 13 | 32.0 (+/- 10.7) | 4 | 9 |
|  | 41 |  |  |  |

## Table S4. Patients used for quantification of sOMGP in the CSF.

| Cohort | Number | Mean age | Male | Female | sOMGP ng/ml |
| --- | --- | --- | --- | --- | --- |
| NINDC | 24 | 40 (+/- 12.3) | 9 | 15 | 151.9 |
| INDC | 13 | 41.9 (+/- 13.4) | 2 | 11 | 164.3 |
| CIS | 17 | 36.4 (+/- 11.8) | 4 | 13 | 151.0 |
| RRMS | 23 | 38.7 (+/- 12.3) | 6 | 17 | 127.7 |
| SPMS | 15 | 51.5 (+/- 9.3) | 6 | 9 | 111.4 |
|  | 92 |  |  |  |  |

Non inflammatory neurological disease control (NINDC), inflammatory neurological disease control (INDC), clinical isolated syndrome (CIS), relapsing remitting multiple sclerosis (RRMS), secondary progressive multiple sclerosis (SPMS)

## Table S5. Animal experiments performed in this study.

| Injected Ab | Injected T cells | | |
| --- | --- | --- | --- |
|  | OVA-specific T cells | MBP-specific T cells | OMGP-specific T cells |
| No Ab | 3 | 0 | 3 |
| 8-18C5-hIgG1 | 3 | 3 | 3 |
| Anti-OMGP | | | |
| 14A9 | 0 | 2 | 0 |
| 31A4 | 0 | 2 | 0 |
| 22H6 | 0 | 2 | 2 |
| 22H6-hIgG1 | 0 | 0 | 3 |
| MAB1674 | 0 | 0 | 3 |
| CTR antibodies | | | |
| IvIg | 3 | 0 | 3 |
| HK3-hIgG1 | 0 | 3 | 3 |
| CTR rIgG2b | 0 | 2 | 0 |
| CTR mIgG2b | 0 | 2 | 0 |
| CTR rIgG2a | 0 | 2 | 2 |
| CTR rIgG1 | 0 | 0 | 3 |

Digits indicate number of used animals.

# SI References

2. Albrecht S, Korr S, Nowack L, Narayanan V, Starost L, Stortz F, Arauzo-Bravo MJ, Meuth SG, Kuhlmann T, Hundehege P (2019) The K2P -channel TASK1 affects Oligodendroglial differentiation but not myelin restoration. Glia 67:870-883. doi:10.1002/glia.23577

9. Brosch M, Yu L, Hubbard T, Choudhary J (2009) Accurate and sensitive peptide identification with Mascot Percolator. J Proteome Res 8:3176-3181. doi:10.1021/pr800982s

18. Emery B, Dugas JC (2013) Purification of oligodendrocyte lineage cells from mouse cortices by immunopanning. Cold Spring Harb Protoc 2013:854-868. doi:10.1101/pdb.prot073973

27. Hauck SM, Dietter J, Kramer RL, Hofmaier F, Zipplies JK, Amann B, Feuchtinger A, Deeg CA, Ueffing M (2010) Deciphering membrane-associated molecular processes in target tissue of autoimmune uveitis by label-free quantitative mass spectrometry. Mol Cell Proteomics 9:2292-2305. doi:10.1074/mcp.M110.001073

45. Mayer MC, Breithaupt C, Reindl M, Schanda K, Rostasy K, Berger T, Dale RC, Brilot F, Olsson T, Jenne D, Pröbstel AK, Dornmair K, Wekerle H, Hohlfeld R, Banwell B, Bar-Or A, Meinl E (2013) Distinction and temporal stability of conformational epitopes on myelin oligodendrocyte glycoprotein recognized by patients with different inflammatory central nervous system diseases. J Immunol 191:3594-3604. doi:10.4049/jimmunol.1301296

49. Misu T, Höftberger R, Fujihara K, Wimmer I, Takai Y, Nishiyama S, Nakashima I, Konno H, Bradl M, Garzuly F, Itoyama Y, Aoki M, Lassmann H (2013) Presence of six different lesion types suggests diverse mechanisms of tissue injury in neuromyelitis optica. Acta Neuropathol 125:815-827. doi:10.1007/s00401-013-1116-7

55. Perera NC, Wiesmuller KH, Larsen MT, Schacher B, Eickholz P, Borregaard N, Jenne DE (2013) NSP4 is stored in azurophil granules and released by activated neutrophils as active endoprotease with restricted specificity. Journal of immunology (Baltimore, Md : 1950) 191:2700-2707. doi:10.4049/jimmunol.1301293

56. Piddlesden SJ, Lassmann H, Zimprich F, Morgan BP, Linington C (1993) The demyelinating potential of antibodies to myelin oligodendrocyte glycoprotein is related to their ability to fix complement. Am J Pathol 143:555-564

57. Pinna D, Corti D, Jarrossay D, Sallusto F, Lanzavecchia A (2009) Clonal dissection of the human memory B-cell repertoire following infection and vaccination. European journal of immunology 39:1260-1270. doi:10.1002/eji.200839129

77. Watkins TA, Emery B, Mulinyawe S, Barres BA (2008) Distinct stages of myelination regulated by gamma-secretase and astrocytes in a rapidly myelinating CNS coculture system. Neuron 60:555-569. doi:10.1016/j.neuron.2008.09.011

78. Weider M, Starost LJ, Groll K, Kuspert M, Sock E, Wedel M, Frob F, Schmitt C, Baroti T, Hartwig AC, Hillgartner S, Piefke S, Fadler T, Ehrlich M, Ehlert C, Stehling M, Albrecht S, Jabali A, Scholer HR, Winkler J, Kuhlmann T, Wegner M (2018) Nfat/calcineurin signaling promotes oligodendrocyte differentiation and myelination by transcription factor network tuning. Nat Commun 9:899. doi:10.1038/s41467-018-03336-3

79. Winklmeier S, Schlüter M, Spadaro M, Thaler FS, Vural A, Gerhards R, Macrini C, Mader S, Kurne A, Inan B, Karabudak R, Ozbay FG, Esendagli G, Hohlfeld R, Kümpfel T, Meinl E (2019) Identification of circulating MOG-specific B cells in patients with MOG antibodies. Neurology(R) neuroimmunology & neuroinflammation 6:625. doi:10.1212/nxi.0000000000000625

80. Wisniewski JR, Zougman A, Nagaraj N, Mann M (2009) Universal sample preparation method for proteome analysis. Nat Methods 6:359-362. doi:10.1038/nmeth.1322
